# Single-cell analysis identified key macrophage subpopulations associated with atherosclerosis

**DOI:** 10.1515/med-2024-1088

**Published:** 2024-12-20

**Authors:** Zhenzhen Zhao, Yuelong Qin, Rui Wu, Wenwu Li, Yujiang Dong

**Affiliations:** First Clinical Medical College, Shandong University of Traditional Chinese Medicine, Jinan, 250000, China; Department of Cardiovascular Disease, The Second Affiliated Hospital of Shandong University of Chinese Medicine, Jinan, 250001, China; Pingyi County Hospital of Traditional Chinese Medicine Cardiology Department, Linyi, 273300, China; Department of Burn Plastic and Wound Repair Surgery, Affiliated Hospital of Youjiang Medical University for Nationalities, Baise, China; Laboratory of the Atherosclerosis and Ischemic Cardiovascular Diseases, Affiliated Hospital of Youjiang Medical University for Nationalities, Baise, China; Shandong University of Traditional Chinese Medicine, Jinan, 250014, China

**Keywords:** atherosclerosis, macrophage, clinical outcome, FCN1, CCL

## Abstract

**Background:**

Atherosclerosis is a lipid-driven inflammatory disease characterized by plaque formation in major arteries. These plaques contain lipid-rich macrophages that accumulate through monocyte recruitment, local macrophage differentiation, and proliferation.

**Objective:**

We identify the macrophage subsets that are closely related to atherosclerosis and reveal the key pathways in the progression of atherosclerotic disease.

**Materials and methods:**

In this study, we characterize the single-cell landscape of atherosclerosis, identifying macrophage subsets closely related to the disease and revealing key pathways in its progression. Using analytical methods like CytoTRACE, Monocle2, Slingshot, and CellChat, we study macrophage differentiation and infer cell trajectory.

**Results:**

The 8,417 macrophages were divided into six subtypes, macrophages: C0 C1QC+ macrophages, C1 SPP1+ macrophages, C2 FCN1+ macrophages, C3 IGKC+ macrophages, C4 FCER1A+ macrophages, C5CALD1+ macrophages. The results of gene set enrichment analysis, Monocle2, and Slingshot suggest that C2 FCN1+ macrophages may play an important role in the progression of atherosclerosis. C2 FCN1+ macrophages interact with endothelial cells via CCL, CXCL, APP, and other pathways to regulate the progression of atherosclerosis.

**Conclusion:**

We identify a key macrophage subgroup (C2 FCN1+ macrophages) associated with atherosclerosis, which interacts with endothelial cells via CCL, CXCL, APP, and other pathways to regulate disease progression.

## 1 Introduction

Atherosclerosis is the main cause of many cardiovascular diseases, such as coronary artery disease, cerebrovascular disease (CVD), and peripheral artery disease, which can lead to myocardial infarction (MI), stroke, amputation, etc. [1,2,3]. MI and stroke are the second most common causes of death after cancer, accounting for 31% of deaths in the United States [4]. Atherosclerosis is triggered by endothelial damage induced by classical risk factors [5] such as high cholesterol, hypertension, obesity, diabetes mellitus, smoking, and low shear stress [6,7], resulting in the localized subendothelial accumulation of low-density lipoproteins (LDLs) susceptible to oxidative modification [8,9]. Specifically, elevated cholesterol levels are a crucial factor in atherosclerosis [10]. The deposition of cholesterol beneath the endothelial layer of blood vessels results in plaque formation, which constitutes the principal pathological alteration in atherosclerosis [8,9]. Smoking inflicts damage on the vascular endothelium and promotes inflammatory responses, which not only accelerates the onset of atherosclerosis but also elevates the risk of cardiovascular events [3,11]. Diabetes increases the risk of cardiovascular disease by inducing endothelial dysfunction and accelerating the progression of atherosclerosis. Individuals with diabetes often present with hyperglycemia, elevated insulin levels, and metabolic syndrome, all of which exacerbate atherosclerosis [12,13]. Atherosclerosis is a lipid-driven inflammatory disease characterized by the formation of plaques in large arteries [12,13]. These fatty plaques contain lipid-rich macrophages that accumulate as a result of the recruitment of circulating monocytes and the local differentiation and proliferation of macrophages [14,15,16]].

Macrophages, as key inflammatory cells, play a central role in the pathogenesis of all stages of atherosclerosis. In the healthy state, the arterial wall contains vascular resident macrophages (Lyve 1 macrophages) in the tunica albuginea, which perform homeostatic functions [17]. Researchers have conducted a large number of studies on the relationship between macrophages and atherosclerosis and found that macrophages play an important role in all stages of atherosclerosis, from the occurrence and expansion of lesions, to the rupture of atherosclerotic lesions through necrosis, and to the regression of atherosclerotic lesions [18,19]. The diversity of macrophage subsets in atherosclerotic plaques demonstrates the plasticity and rich biological functions of this cell type [6,20]. Macrophages in atherosclerosis exhibit distinct phenotypes and functions, reflecting their heterogeneity across various stages of the disease and within different microenvironments. Both M1 macrophages and M2 macrophages are closely related to inflammatory responses, among which M1 macrophages are mainly involved in pro-inflammatory responses and M2 macrophages are mainly involved in anti-inflammatory responses [21,22,23]. The implementation of new methods for extended multidimensional analysis has broadened our understanding of the cellular diversity present in atherosclerotic plaques [24,25]. An intermediate step in expanding the number of antigens that can be assessed on individual cells was the development of mass spectrometry cytometry [26], which allows the assessment of ∼35–42 surface markers [27], including intracellular cytokines or transcription factors, but does not provide information on mRNA [4].

Advances in single-cell technology have enabled high-dimensional transcriptomic and proteomic profiling of individual blood and tissue cells, as well as the use of clinical models to predict clinical treatment responses [28,29]. Recent single-cell studies have revealed new cellular heterogeneity of atherosclerotic plaque tissues and allowed a better understanding of the different immune function states in the context of atherosclerosis. Fundamentally changing our ability to characterize the cellular heterogeneity of blood and tissue specimens. Unlike bulk RNA sequencing, which averages cellular transcriptional expression, single-cell RNA sequencing (scRNA-seq) quantifies gene expression in individual cells in both normal and pathological settings [30,31]. Conventional bulk RNA sequencing methods typically involve sequencing a mixture of cells, thereby masking the heterogeneity among different cell types. However, in the context of atherosclerosis research, understanding the functional characteristics and interactions of distinct cell types is crucial for disease progression. Atherosclerosis is a heterogeneous disease, with different cell types playing distinct roles in its pathogenesis. ScRNA-seq captures this cellular heterogeneity, aiding our understanding of the functional characteristics and interactions of various cell types during disease development. ScRNA-seq reveals the activity and alterations of intracellular signaling pathways, facilitating our comprehension of the molecular mechanisms underlying atherosclerosis. By analyzing changes in key pathways, we can identify novel therapeutic targets and strategies.

**Figure 1:**
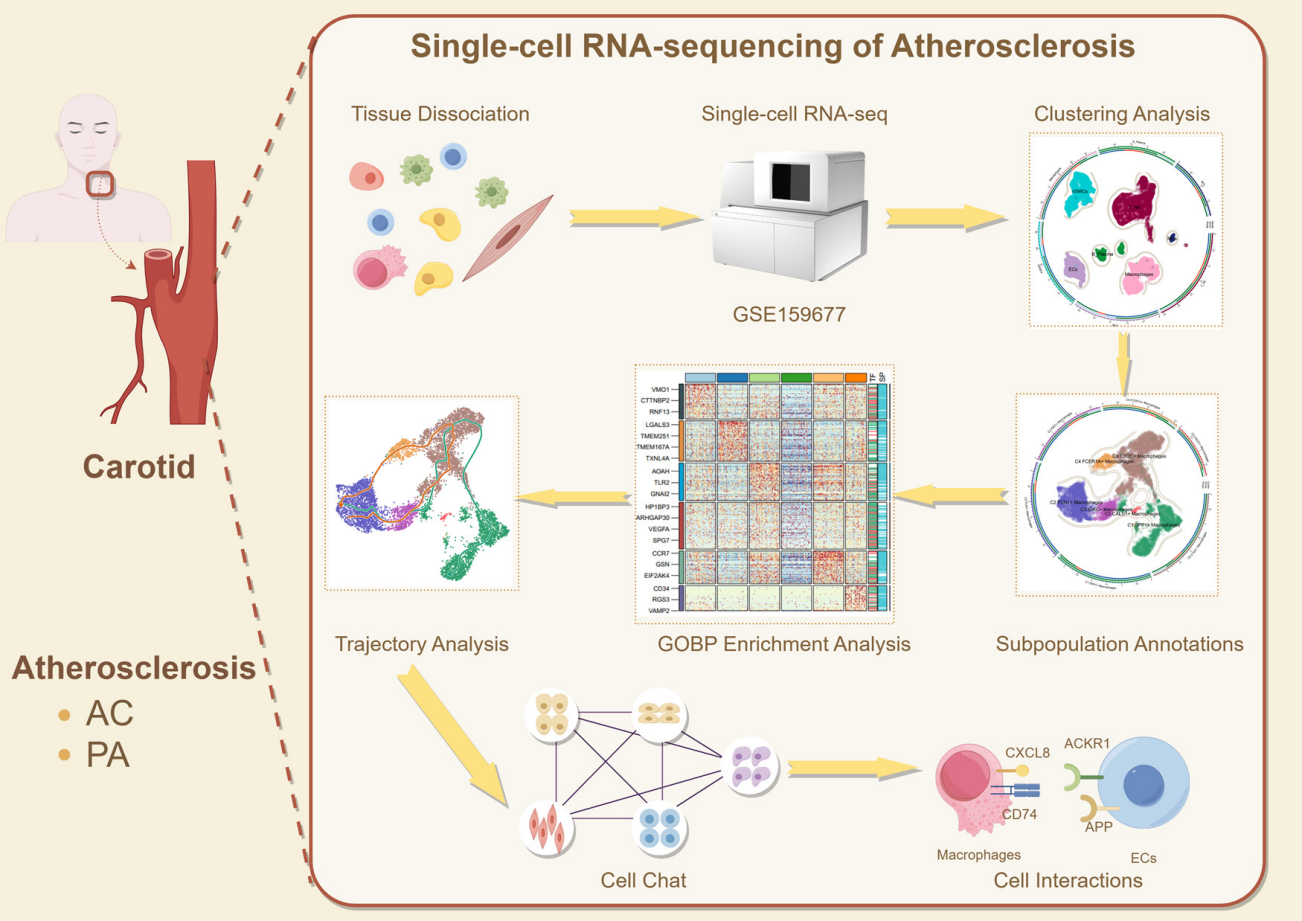
The flow diagram of this study. The biological characteristics of macrophages in carotid atherosclerosis were studied by single-cell analysis methods such as dimension reduction, clustering, pseudotiming, and cell communication. C2 FCN1+ macrophages interacted with endothelial cells through the APP and CXCL signaling pathways. The most active receptor–ligand pairs were APP–CD47 and CXCL8–ACKR1.

In this article, we investigate macrophage differentiation processes in atherosclerosis by characterizing the single-cell landscape of atherosclerosis, using a range of analytical methods for single-cell sequencing, such as CytoTRACE, Monocle2, Slingshot, and CellChat, to infer cellular trajectories and identify macrophage subpopulations that are closely associated with atherosclerosis. To study the cellular functions of key macrophage subpopulations and reveal the key pathways in the progression of atherosclerosis, we hope to provide new therapeutic targets for better clinical guidance and also play an important role in the research of single-cell technology in the field of cardiovascular diseases [32] (Figure 1).

## 2 Materials and methods

### 2.1 Single-cell data acquisition and processing

scRNA-seq data of atherosclerotic cells were obtained from the Gene Expression Omnibus database (GSE159677). The original sequencing data from 10× Genomics were compared and quantified using CellRanger (10× Genomics) suite (version 3.0.2). The original gene expression matrix was introduced into R software (version 3.6.3) using the Seurat R software package (version 3.1.5) [33,34]. We use the “Double-tFinder” algorithm to identify cells and rigorously control the quality of scRNA-seq data. Cells that met the following criteria were not included: The reading segments with a total number of molecules (nCount_RNA) greater than 100,000 or more than 25% were mapped to mitochondria or more than 5% to red blood cells, or less than 300 unique genes (nFeature_RNA) were expressed, more than 6,000 unique genes were expressed, or fewer than 500 molecules were detected in each cell. Because the data we use come from publicly available databases, this study does not need ethical approval.

### 2.2 Identification of differentially expressed genes and subgroups

Using Seurat 2.3, the expression matrix was first processed by the function “NormalizedData” normalize [35]. Using the normalized expression matrix and “FindVariableFeatures” function, the top 2,000 were screened out HVG (hypervariable gene) [36,37]. We then normalized the dataset using the “ScaleData” function. We use the “Harmony” software package (version 0.1.0) to correct the batch effect between data sets [38,39]. Dimensionality reduction was conducted using the “RunPCA” function, with the first 30 valid principal components selected. Using clustering analysis with the “FindNeighbors” and “FindClusters” functions [34,40]. We use the “FindAllMarkers” function to determine the differential genes (marker genes) of each cluster [41,42,43]. Annotate according to “CellMarker” and “singleR” software and check it manually to make sure it is used, so as to ensure the correct result.

### 2.3 Enrichment analysis of differential genes by GOBP and gene set enrichment analysis (GSEA)

The top 100 upregulated DEG of each cluster uses the clusterProfiler R package to perform gene ontology (GO) analysis [44]. This study focuses on the functional gene set of biological processes (BPs). The *q* value was used to select significantly rich results with a critical value of 0.05. The results of GO enrichment analysis were filtered according to *Q* value (*Q*valueCutoff = 0.05). GO analysis [45,46,47] identified its molecular function (MF), cellular components (CCs), BP, and related signal pathways [48,49,50].

GSEA was a computational method for determining whether an *a priori*-defined set of genes shows statistically significant and consistent differences between control and experimental groups [51,52,53]. Differential genes were ranked according to signature pathways described in a molecular characterization database to identify the pathways that differ most between subgroups. These genes were ranked from highest to lowest fold difference and analyzed by GSEA [54].

### 2.4 Pseudo-time analysis

CytoTRACE (Cell Trace Reconstruction Analysis Using Gene Counting and Expression [Cyto]) [55] was a computational method that predicts the relative state of differentiation of cells based on single-cell RNA sequencing data. CytoTRACE was developed to predict the state of differentiation in the scRNA-seq data without any prior information.

Pseudo-time locus of atherosclerotic macrophages was inferred by using the Monocle pseudo-time pedigree locus, and the pseudo-time dependent gene was expressed visually [56]. Different macrophage cell subsets were arranged according to pseudo-time series, and a pseudo-time thermogram was used to identify and display genes that change at the same time as pseudo-time [57].

Slingshot was a method to infer cell lineage and false time from single-cell gene expression data. It was a unique robust and flexible tool, which combines the highly stable technology required by noisy single-cell data and the ability to identify multiple trajectories. Accurate pedigree inference was the key step to identify dynamic time gene expression [58]. The cell lineage was inferred by fitting the minimum spanning tree with the getLineages function and estimated by the getCurves function. We mapped the differentiation trajectories of each subcluster and assessed the dynamic changes in gene expression over time.

### 2.5 Cell communication

CellChat [59] uses network analysis and pattern recognition methods to predict the main signal input and output of cells and how these cells and signals coordinate their functions. Through a variety of learning and quantitative comparisons, CellChat classifies signal paths and describes conservative and context-specific paths in different data sets.

### 2.6 Statistical analysis

All statistical analysis and graph generation were performed in R (version 3.6.3). Students’ *T*-tests were used to analyze data.

## 3 Results

### 3.1 Sample sources and quality control for single-cell analysis

To investigate the major cell types involved in the progression of atherosclerosis, we collected atherosclerotic samples from three patients for single-cell sequencing analysis. Principal component analysis was used to select the most important 30 principal components and were used for UMAP downscaling (Figure S1a–d). For the AS cells' nFeature_RNA, nCount RNA, pMT, pHB, and pRP levels were tightly controlled, and the 45,690 high-quality cells were ultimately retained (Figure S1e–g). The differential genes of these cells were clustered in a dimensionality-decreasing manner, yielding 29 cell clusters, and we annotated 34 cell clusters with labeling by typical markers defined in the literature (Figure S1h and i).

We explored single-cell RNA-seq data from atherosclerosis using inferCNV to identify cells with chromosomal mutations in atherosclerosis by inferCNV. Based on single-cell transcriptome data, we identified macrophage subpopulations with abnormal chromosome copy number amplification or deletion using inferCNV. C0 C1QC+ macrophages, C2 FCN1+ macrophages, and C4 FCER1A+ macrophages had high chromosome copy number variation, which may be related to the progression of atherosclerosis (Figure S2a).

### 3.2 Single-cell analysis of major cell types in atherosclerosis

**Figure 2:**
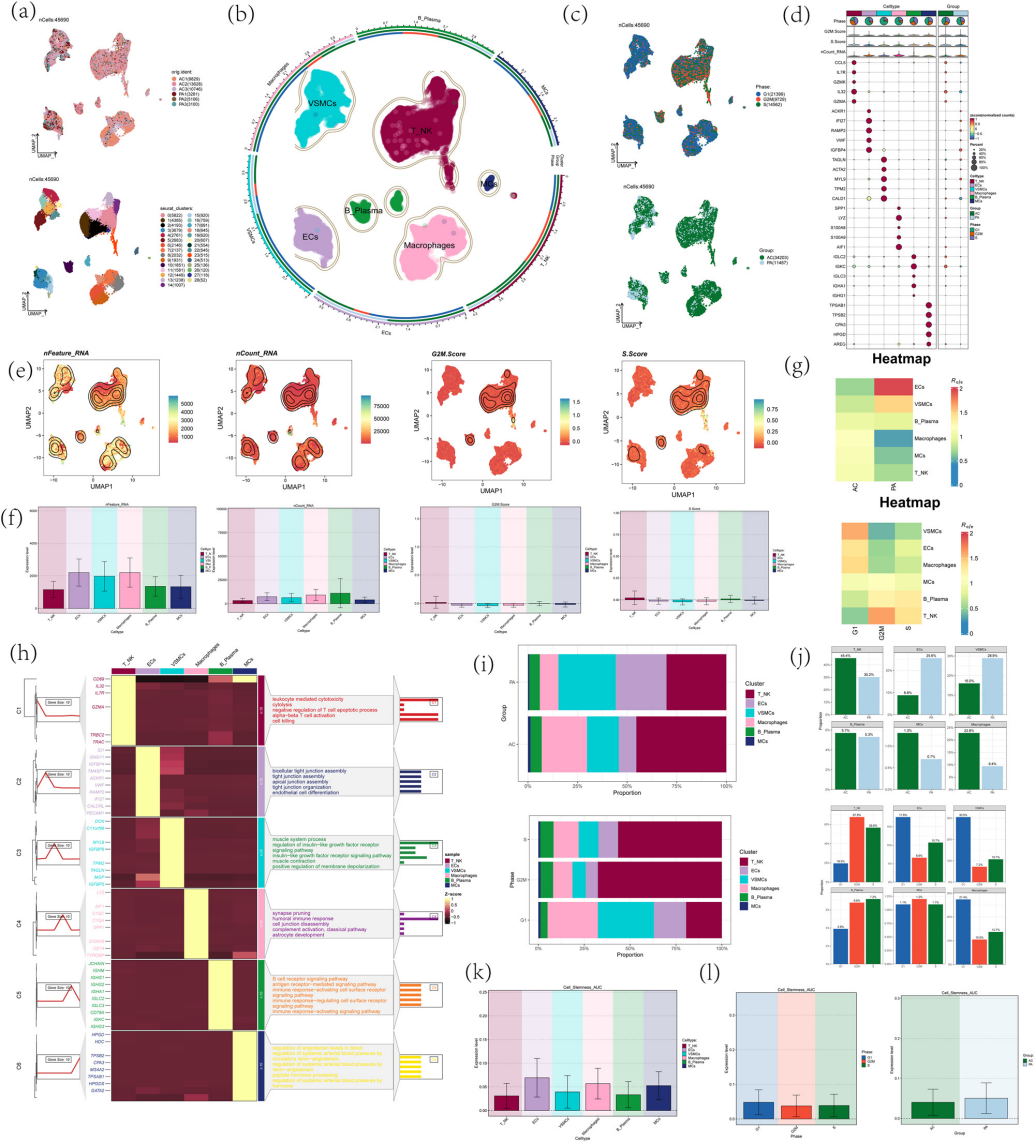
Identification of major cell types in atherosclerotic disease. (a–c) UMAP plot was used to demonstrate the distribution of 45,690 high-quality cells in terms of sample source, downscaled clustering (serurat method of clustering), tissue site (AC, atherosclerotic core; PA, adjacent portion), and cell cycle (G1, G2M, S), and annotated into 6 cell types based on differential gene expression. (d) Bubble plots demonstrating the differential expression of the top five most significant (top5) maker genes for the six cell types and different tissue types. The color of the bubbles indicates the expression and the size indicates the percentage of expression. (e) Umap plot demonstrating the distribution of nCount_RNA, nFeature_RNA, S.Score, and G2M.Score for the six cell types. (f) Bar plot demonstrating the difference levels of nCount_RNA, nFeature_RNA, S.Score, and G2M.Score for the six cell types. (g) Heatmap demonstrating the tissue origin of the 6 major cell types of atherosclerosis and cell cycle distribution. (h) Heatmap showing the results of enrichment analysis of differential genes of the six major cell types. (i and j) Stacked bar graphs and faceted graphs showing the percentage of the six major cell types in different tissue sites and cell cycles. (k and l) Bar graphs showing the differential expression levels of stemness genes in different cell types, tissue sources, and cell cycles.

UMAP plots were used to demonstrate the distribution of sample source, downscaling clustering (serurat method of clustering), tissue site (CA: atherosclerotic core; PA: adjacent portion), and cell cycle (G1, G2M, S) of 45,690 high-quality cells obtained after preliminary quality control and annotated into six cell types based on differential gene expression, including T_NK, ECs, VAMCs, macrophages, B_Plasma, and MCs (Figure 2a–c). The expression of the top five most significant (top5) differential genes among the six cell types was shown in Figure 2d. UMAP plots and violin plots were demonstrated in atherosclerotic samples by the distribution of nCount_RNA, nFeature_RNA, S.Score, and G2M.Score in the six major cell types shown, with higher nFeature_RNA levels in ECs, VAMCs, and macrophages; B_Plasma had higher nCount_RNA levels; T_NK cells were more predominantly in the G2M phase, which was in an active state of cellular proliferation (Figure 2e and f). Cell abundance plots (Figure 2g) and stacked bar graphs (Figure 2i and j) demonstrated the tissue origin and cell cycle distribution of the six major cell types of atherosclerosis, ECs, VAMCs mainly originated from PA, macrophages, T_NK mainly originated from the AC, B_Plasma, and MCs accounted for a smaller proportion and originated from the atherosclerotic core and neighboring sites; T_Plasma and MCs were less and originated from the AC and PA. Differential gene enrichment analysis of the six major cell types showed that T_NK cells were enriched in leukocyte-mediated cytotoxicity, cytolysis, negative regulation of T-cell apoptotic process, alpha-beta T-cell activation, cell killing, and T_NK cell activation and cell killing; ECs were enriched in bicellular tight junction assembly, tight junction assembly, apical junction assembly, tight junction organization, and endothelial cell differentiation; VAMCs were enriched in muscle system process, regulation of insulin-like growth factor receptor, signaling pathway, and insulin-like growth factor receptor (Figure 2h). Higher expression levels of stemness genes were found in ECs and macrophages, as well as in the G1 cycle, and relatively higher expression levels of stemness gene sets in PA of atherosclerosis (Figure 2k and l).

### 3.3 Differential analysis of T cells and NK cells in atherosclerosis

CD4+ T cells were frequently present in atherosclerotic plaques. A substantial body of evidence suggests that T helper 1 (TH1) cells exhibit pro-atherogenic properties, while regulatory T (Treg) cells demonstrate anti-atherogenic functions; however, Treg cells can also adopt pro-atherogenic roles [60]. Cholesterol was a component of the TCRβ subunit, which forms part of the TCR complex. Cholesterol and sphingomyelin create lipid rafts (nanodomains) at the surface of T cells (TCR). Understanding the heterogeneity and functional characteristics of T-cell subsets was crucial for further elucidating the progression of atherosclerotic disease. Thus, we conducted an analysis of the various features of T-cell and NK cell subsets. These cells were categorized into six subsets: proliferative CD8 T, NK, CD4 naive T, CD8 effector T, CD4 effector T, and Treg (Figure S3a). The expression of the top five differential genes among these six subsets was illustrated in Figure S3b, where RPS3A, RPL23, and RPL22 exhibit relatively high expression levels. The stacked bar chart demonstrates the tissue origin proportions of T-cell and NK-cell subsets, revealing that in the atherosclerotic condition (AC), CD4 effector T cells and CD8 effector T cells were more prevalent, whereas in the plaque area (PA), CD8 effector T cells and Treg cells dominate (Figure S3c). Subsequently, we analyzed the tissue origin and cell cycle distribution of T-cell and NK-cell subsets, finding that proliferative CD8 T cells, CD4 effector T cells, and Treg cells were more abundant in AC than in PA (Figure S3d and e). Proliferative CD8 T cells exhibited higher nCount_RNA, nFeature_RNA, G2M.Score, and S.Score compared to other cell subsets (Figure S3f and g). Finally, we performed enrichment analysis on the differential genes among these subsets, with upregulated and downregulated genes presented in a volcano plot (Figure S3h). GSEA results indicated that CD4 naive T cells were enriched in cytoplasmic translation and translation pathways; CD4 effector T cells were enriched in cellular response to oxidative stress and blood vessel development pathways; CD8 effector T cells were enriched in peptide antigen assembly with MHC protein complex and MHC protein complex assembly pathways; NK cells were enriched in cell killing and innate immune response pathways; proliferative CD8 T cells were enriched in regulation of mitotic sister chromatid segregation and kinetochore organization pathways; and Treg cells were enriched in tumor necrosis factor-mediated signaling pathway and T-cell homeostasis pathways (Figure S3I). Enrichment analysis results for GOBP terms show that proliferative CD8 T cells were enriched in chromosome segregation, sister chromatid segregation, and mitotic nuclear division pathways, while Treg cells were enriched in regulation of T-cell activation, leukocyte cell–cell adhesion, and regulation of leukocyte cell–cell adhesion pathways (Figure S3J).

### 3.4 Identification and visualization of atherosclerotic macrophage subsets

**Figure 3:**
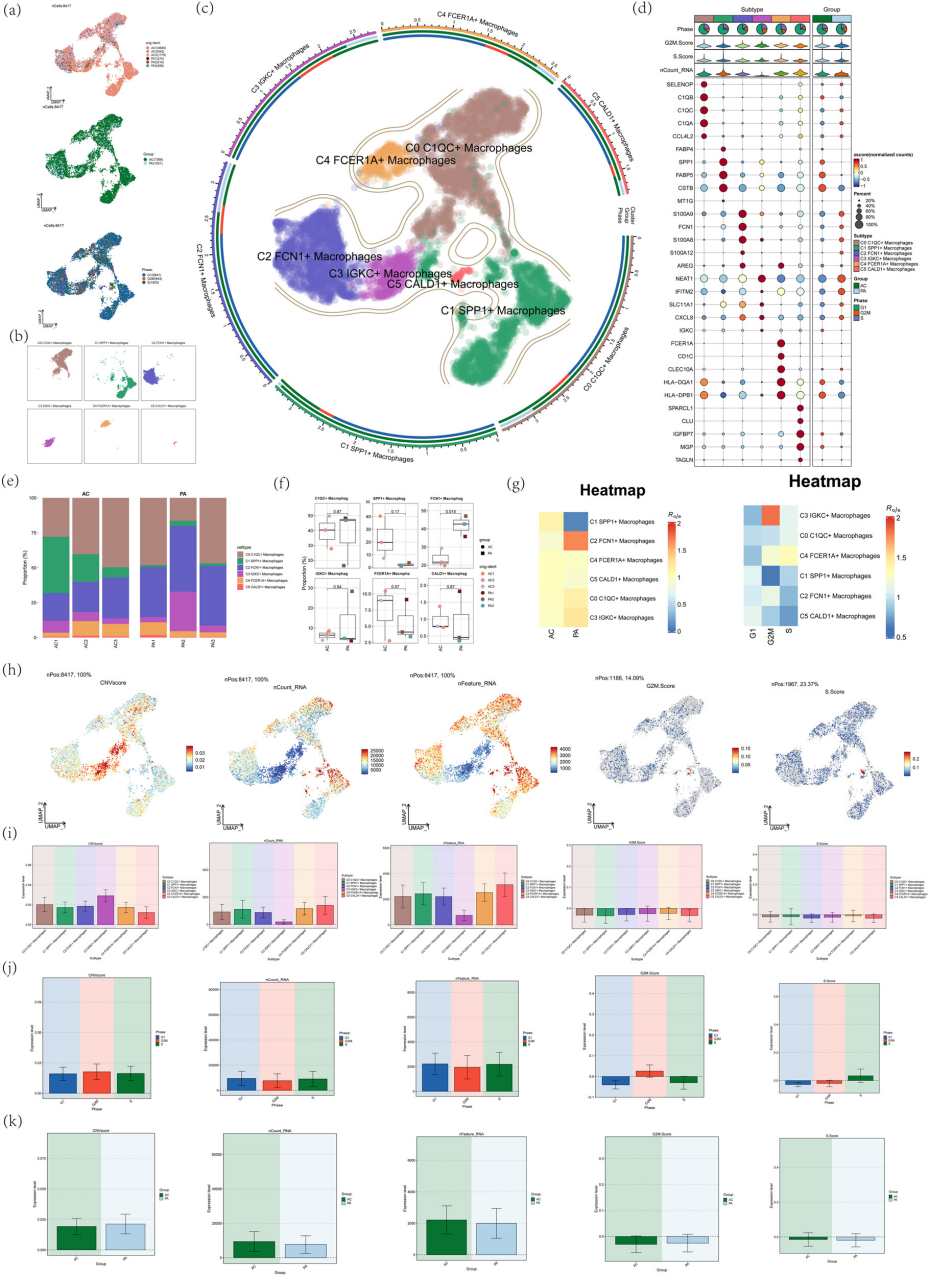
Atherosclerotic macrophage subpopulations. (a) UMAP plots of 8417 high-quality atherosclerotic macrophage samples source, tissue type (AC, PA), and cell cycle (G1, G2M, S). (b and c) UMAP plots were used to demonstrate the unsupervised clustering of atherosclerotic macrophages into six cell clusters (C0 C1QC+ macrophages, C1 SPP1+ macrophages, C2 FCN1+ macrophages, C3 IGKC+ macrophages, C4 FCER1A+ macrophages, C5CALD1+ macrophages). (d) Bubble plots showing the expression of the 5 most significant differential genes (top5) in macrophage subpopulations as well as in different tissue types. (e–g) Stacked bar graphs, box line graphs, and cell abundance graphs demonstrating the relative abundance (as a percentage) of the six macrophage subpopulations in different tissue types and in the cell cycle. (h) Distribution of CNVscore, nCount_RNA, nFeature_RNA, G2M.Score, and S.Score of the six macrophage subpopulations using UMAP plots. (i) Bar plots demonstrating the levels of CNVscore, nCount_RNA, nFeature_RNA, G2M.Score, and S.Score for different macrophage subpopulations. (j and k) Bar plots demonstrating CNVscore, nCount_RNA, nFeature_RNA, G2M.Score, and S.Score levels for different tissue types and cell cycles.

After analyzing the atherosclerotic macrophages, 8,417 high-quality macrophages were obtained after quality control and batch effect removal. Umap diagram shows the results of removing the cell source and batch effect of three samples of atherosclerosis (upper part of Figure 3a) and shows the tissue types and cell cycle distribution of these high-quality cells (lower part of Figure 3a). Next, six different cell clusters were obtained by further dimensionality reduction clustering. According to the maker genes of various cells, these high-quality macrophage clusters were annotated into six kinds of macrophage clusters, including C0 C1QC+ macrophages, C1 SPP1+ macrophages, C2 FCN1+ macrophages, C3 IGKC+ macrophages, C4 FCER1A+ macrophages, and C5 CALD1+ macrophages (Figure 3b and c). The differential expression of the maker gene (top5) in six cell types and tissues was shown in Figure 3d. Next, stacked bar charts, box charts, and thermal maps (cell abundance maps) were used to show the relative abundance (proportion) of six macrophage subsets in different tissue types and cell cycles (Figure 3e–g). C0 C1QC+ macrophages account for more in AC and PA, and C1 SPP1+ macrophages account for more in AC. C2 FCN1+ macrophages account for a large proportion in both AC and PA and a relatively large proportion in PA, C3 IGKC+ macrophages account for a large proportion in PA, and C4 FCER 1A+ macrophages and C5 Cald1+ macrophages account for a small proportion in both AC and PA. It was suggested that C0 C1QC+ macrophages, C1 SPP1+ macrophages, and C2 FCN1+ macrophages may play an important role in the progression of atherosclerosis. To further clarify the differences of atherosclerotic macrophage subsets, we studied the overall distribution of CNVscore, nFeature_RNA, nCount _RNA, S.Score, and G2M.Score in each macrophage subset (Figure 3h) and further showed their average expression levels by histogram (Figure 3i). The CNVscore of C0 C1QC+ macrophages and C3 IGKC+ macrophages was higher. It shows that there were many chromosome copy number variations, and gene mutation may occur. The elevated scores of nFeature_RNA and nCount_RNA in C1 SPP1+ macrophages, C2 FCN1+ macrophages, C4 FCER1A+ macrophages, and C5 CALD1+ macrophages indicate that these cell subsets were more active in atherosclerotic disease. The above indicates that these cell subsets are in a relatively active state of cell proliferation. Finally, we analyzed the atherosclerotic macrophages. There were some differences in the levels of CNVscore, nFeature_RNA, nCount _RNA, S.Score, and G2M.Score in different tissue types and different cell cycles. The scores of nFeature_RNA and nCount _RNA of AC were higher, and the score of CNVscore of PA was higher (Figure 3j and k).

### 3.5 Enrichment analysis of macrophages in atherosclerosis

**Figure 4:**
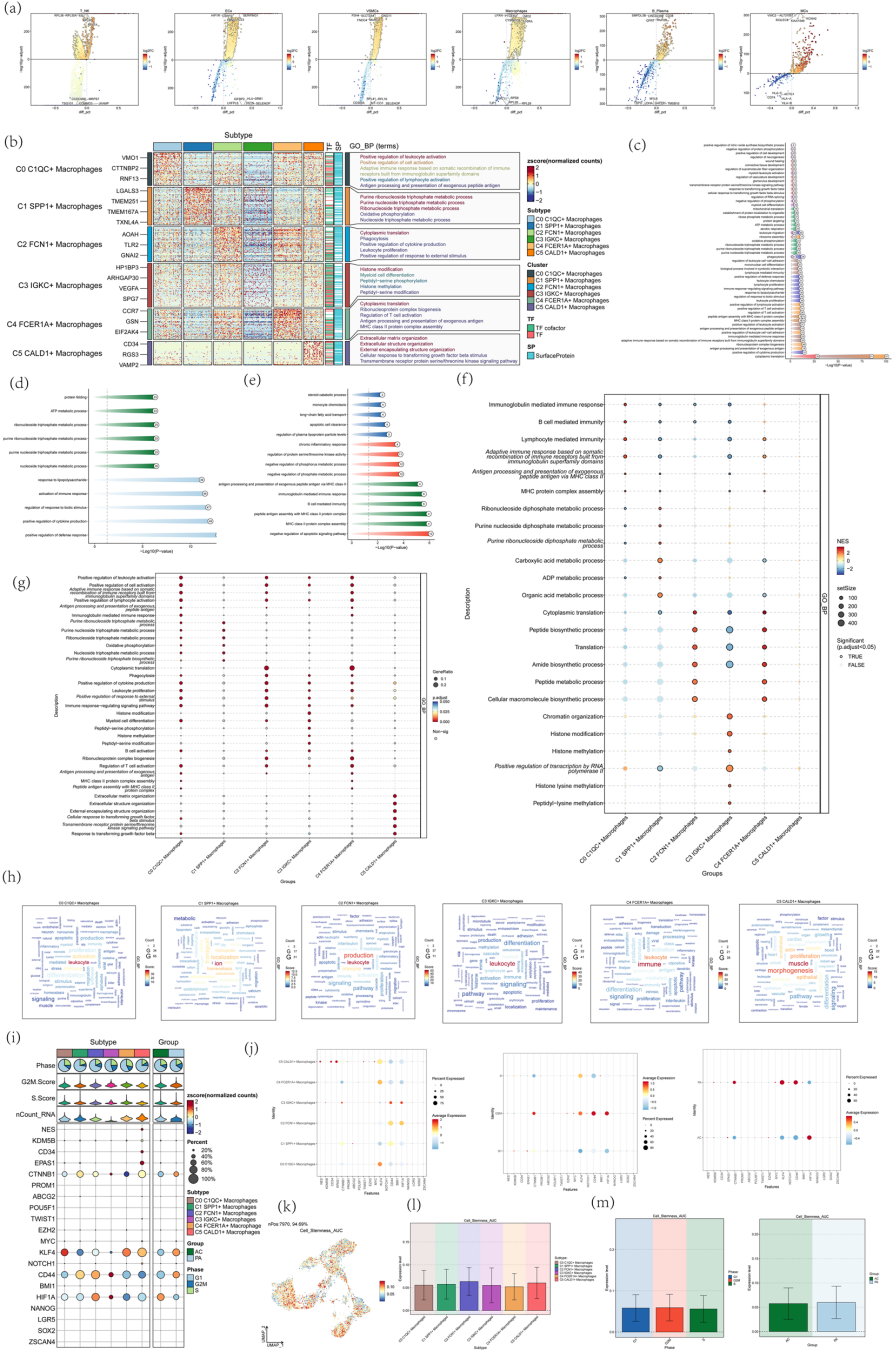
Atherosclerosis macrophage enrichment analysis results. (a) Volcano plot of differentially expressed genes in six macrophage subtypes. (b) Heatmap showing the first 5 enrichment entries of GOBP enrichment analysis of macrophage subtype differential genes. (c–e) Atherosclerotic macrophages in different cell types, tissue origin, and cell cycle enrichment analysis results. (f) Results of macrophage subtype differential gene enrichment analysis based on GOBP entries after GSEA scoring were shown by bubble plots. Bubble size indicates GeneRatio, and color indicates scoring by GSEA. (g) Results of the fibroblast subtype differential gene enrichment analysis based on GOBP entries were shown by bubble plots. Bubble size indicates GeneRatio and color represents P.adjust. (h) Word cloud plot of macrophage subtype differential gene GOBP enrichment analysis. (i) Differential expression of stemness genes in macrophage subtypes was shown using bubble plots. (j) Bubble plot demonstrating differential expression of stemness gene sets in different macrophage subtypes, tissue sources, and cell cycle. (k) UMAP plot demonstrating differential expression of stemness genes scored by AUC. (l and m) Bar plots demonstrating differential expression of stemness gene sets across macrophage subtypes, tissue sources, and the cell cycle.

First, we screened the different bases of six kinds of fibroblast subsets and represented them with volcanic maps (Figure 4a) for enrichment analysis of these differential genes. To understand the enrichment of genes in the gene set of atherosclerosis macrophage subpopulation in terms of BP, MF, and cell composition, we analyzed the GOBP enrichment analysis results of six macrophage differential genes and further demonstrated the enrichment results of macrophages in different tissue sources and cell cycles (Figure 4b–e). C0 C1QC+ macrophages are enriched in pathways such as positive regulation of leukocyte activation, positive regulation of cell activation, and lymphocyte-mediated immunity pathways; C1 SPP1+ macrophages were enriched in pathways such as purine ribonucleoside triphosphate metabolic process, oxidative phosphorylation, and carboxylic acid metabolic process; C2 FCN1+ macrophages were enriched in pathways such as cytoplasmic translation, phagocytosis, and translation; C3 IGKC+ Macrophages were enriched in pathways such as Histone modification, Histone methylation, and chromatin organization; C4 FCER1A+ macrophages were enriched in pathways such as ribonucleoprotein complex biogenesis, and regulation of T cell activation; C5 CALD1+ macrophages were enriched in pathways such as extracellular matrix organization, extracellular structure organization, and external encapsulating structure organization (Figure 4f and g). Finally, the word cloud map showed the enrichment of six macrophage differential genes in different pathways (Figure 4h). To study the expression of dry gene set in atherosclerotic macrophages and understand the differentiation potential of different macrophage subsets, we analyzed the expression of related dry genes and showed the expression level of dry genes through bubble maps. We compared the expression level of dry genes in six types of macrophage subsets, different tissue types, and different cell cycles. As shown in Figure 4i and j, dry genes with relatively high expression levels included CTNNB1, KLF4, CD44, and HIF1A. Finally, UMAP and bar graph were used to show the differential expression of the dry gene macrophages scored by AUC. C2 FCN1+ macrophages have a higher dry gene set score, indicating its high differentiation potential, and may play an irreplaceable role in disease progression (Figure 4k–m).

### 3.6 Visualization of CytoTRACE and Monocle2 analysis results in atherosclerotic macrophages

**Figure 5:**
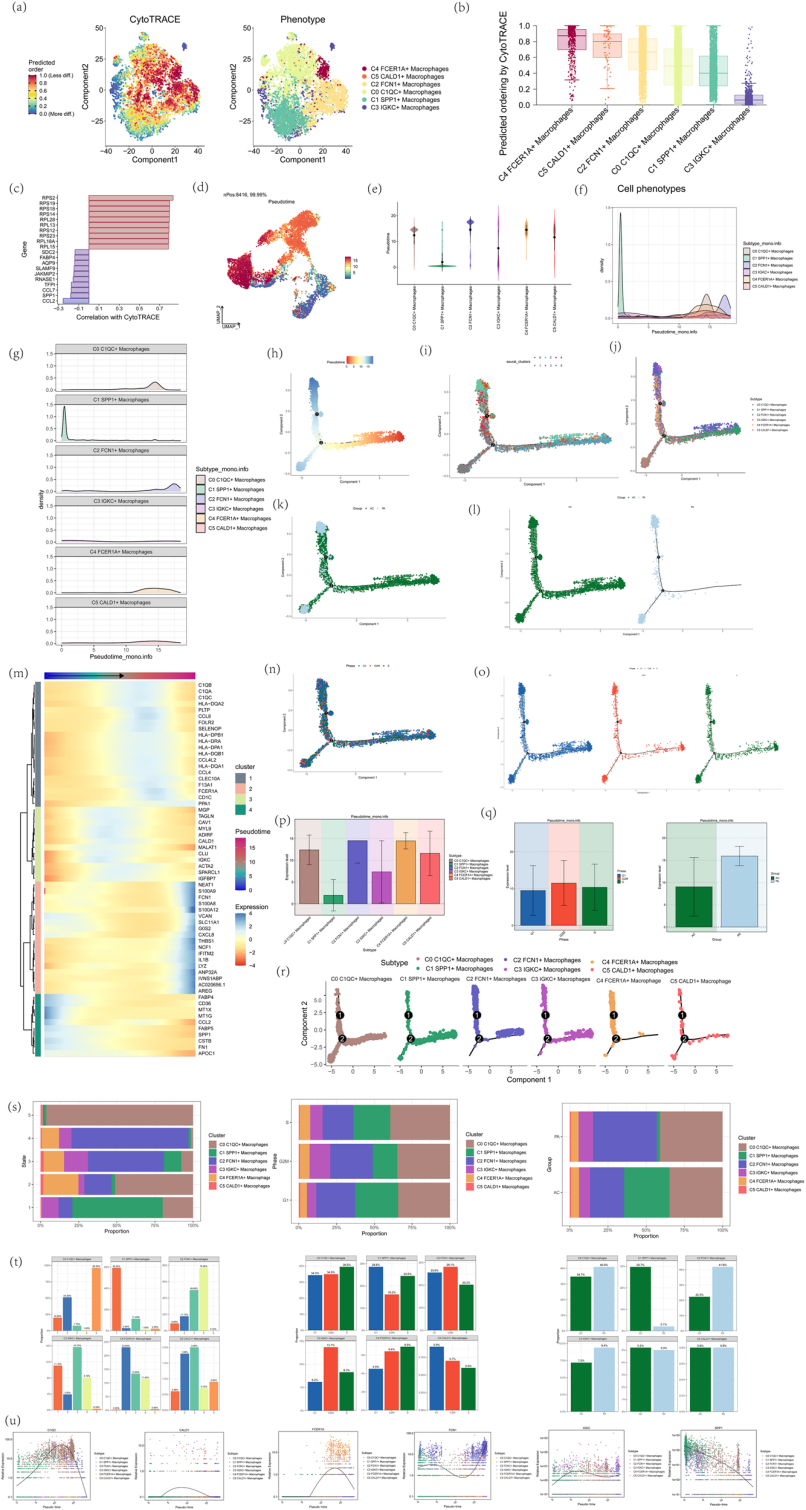
Cytotrace and monocle2 trajectory analysis reveal different differentiated states of atherosclerotic macrophages. (a) The differentiation ability of atherosclerotic macrophages was analyzed using cytotrace (left), where redder colors represent higher stemness and higher differentiation potential and bluer colors represent lower stemness and lower differentiation potential; macrophages were colored according to their different cell types to demonstrate the differentiation potential of macrophages (right). (b) Box-and-line graphs demonstrating the cellular stemness levels of the six macrophage species. (c) Bar graph demonstrating the correlation of stemness genes in the cytotrace analysis, with red indicating positive correlation and purple indicating negative correlation. (d–f) UMAP plot demonstrating the distribution of the proposed temporal trajectories analyzed using Monocle. Blue color shows the starting point of the proposed temporal trajectory and red color shows the end point of the proposed temporal trajectory. Violin and ridge plots demonstrate the distribution of the six macrophage types with the proposed temporal trajectories. (g) Fractional ridge plot further demonstrates the distribution of macrophages in the proposed temporal trajectory. (h–j) UMAP plot demonstrating the proposed temporal trajectory of atherosclerotic macrophages. The right side was the starting point, with two branches to the right, one of which was like the lower left and one of which was divided into two more branches to the upper left. (k and l) UMAP plot shows the distribution of AC and PA tissue types in the proposed temporal trajectory of monocle2. AC: atherosclerotic core, PA: adjacent site. (m) The heatmap shows the differential expression of target genes with pseudo time series. (n and o) UMAP plots demonstrate the distribution of different cell cycles in the monocle2 proposed temporal trajectory. (p and q) Bar plots further demonstrate the expression of different cell types, different tissue distributions, and the distribution of different cell cycles with cell trajectories. (r) Two-dimensional trajectory plots demonstrating the distribution of each of the six types of macrophages with the trajectories. (s) Stacked bar graphs show the percentages of different macrophages, different tissue sources, and different cell cycles in each of the five cell trajectory phases. (t) The segmented bar graphs show the percentages of different macrophages, different tissue sources, and different cell cycles in the five cell trajectory phases for different macrophage tissue sources. (u) Scatter plots show the expression of macrophage maker genes in chronological order.

To infer the differentiation trajectory of atherosclerotic macrophages, we used CytoTRACE and Monocle2 to analyze these cells. CytoTRACE utilizes a probabilistic model to estimate the progression of cells along a trajectory and identify key genes driving cell fate decisions. It focuses on capturing dynamic changes and lineage relationships within a cell population. Monocle2 employs a reverse graph embedding approach to order cells along a trajectory, allowing the visualization of continuous developmental processes. It also provides tools for differential expression analysis and identification of genes driving cell fate decisions. The differentiation ability of atherosclerotic macrophages was analyzed using CytoTRACE, the differentiation potential of macrophages was demonstrated by coloring the macrophages according to their different cell types (Figure 5a), and the cell stemness levels of the six types of macrophages were further demonstrated by a box-and-line plot to speculate on the cell differentiation trajectories (Figure 5b). The correlation of the stemness genes in the CytoTRACE analysis was shown in Figure 5c. Next, the R software Monocle package (version 2.22.0) was applied to assess the differentiation of atherosclerotic macrophage cell subtypes, to understand the differentiation kinetics of different macrophage subpopulations in atherosclerosis, and to infer the dynamic changes during differentiation. The metastatic trajectories of the cells showed different clusters in pseudotimes, as shown in Figure 5d–g, and the proposed temporal distribution of macrophage subpopulations was significantly differentiated, with the lowest proposed temporal scores for C1 SPP1+ macrophages, which were at the initial stage of differentiation, and the highest proposed temporal scores for C2 FCN1+ macrophages, which were at the terminal stage of differentiation. We showed the process of macrophage differentiation with the trajectory by two-dimensional trajectory diagrams, respectively, showing the proposed temporal order predicted by the monocle, the distribution of the five states, and the distribution of cell types with the proposed temporal order under the trajectory, in which it can be inferred that there were two branches in the process of atherosclerotic macrophage cell differentiation, with the right side as the starting point, and two branches to the right, in which one was like a downward left side, and a upward to the left was further divided into two branches, C1 SPP1+ macrophages were located at the initial position of the proposed temporal trajectory, and C2 FCN1+ macrophages were located at the start and end points of the proposed temporal trajectory (Figure 5h–j), and the two-dimensional trajectory faceted Figure 5r further demonstrated the distribution of the six types of macrophages with the proposed temporal trajectory. Then, UMAP plots were used to further demonstrate the distribution of the two tissue types AC and PA (Figure 5k and l) and different cell cycles (Figure 5n and o) in the proposed temporal trajectory of monocle2, and bar graphs were used to further visualize the above results (Figure 5p and q). Next, bar graphs were used to demonstrate the distribution of different macrophage subpopulations in each of the five states, C1 SPP1+ macrophages that were mostly in state1, C0 C1QC+ macrophages were mostly in state5, C2 FCN1+ macrophages, C3 IGKC+ macrophages that were mostly in state3 and state4, C4 FCER1A+ macrophages, and C5CALD1+ macrophages that were mostly in state2 and state3. It can be roughly inferred that atherosclerotic macrophages undergo an evolution from C1 SPP1+ macrophages to C4 FCER1A+ macrophages, C5CALD1+ macrophages, C2 FCN1+ macrophages, C3 IGKC+ macrophages, and finally, C0 C1QC+ macrophages. The percentage of the six types of macrophages in different cell cycles did not vary much (Figure 5s and t). Finally, the heatmap shows the expression changes of macrophage differential genes with the proposed time sequence (Figure 5m), and the scatter plot shows the expression curve of macrophage maker genes with the time sequence (Figure 5u). In summary, C2 FCN1+ macrophages were predominantly distributed in the initial and terminal stages of the pseudotemporal sequence. During the early stage, endothelial cell damage leads to disruption of the vascular endothelial barrier, prompting monocytes from the bloodstream to migrate to the subendothelial layer and differentiate into macrophages. These macrophages were recruited to the site of damage through the expression of specific chemokine receptors (such as CCR2) and cell adhesion molecules [61]. In the later stages of plaque development, the number of M2 macrophages typically increases. These cells secrete anti-inflammatory factors (such as IL-10 and TGF-β), which help alleviate local inflammation and promote plaque stability [62]. Therefore, we propose that C2 FCN1+ macrophages represent a crucial cell subset in the progression of atherosclerotic disease, facilitating macrophage recruitment and lipid accumulation in the early stages, and potentially contributing to plaque stabilization through anti-inflammatory actions or promoting thrombosis during plaque rupture in the later stages.

### 3.7 Slingshot analysis of cellular trajectories of atherosclerotic macrophages

**Figure 6:**
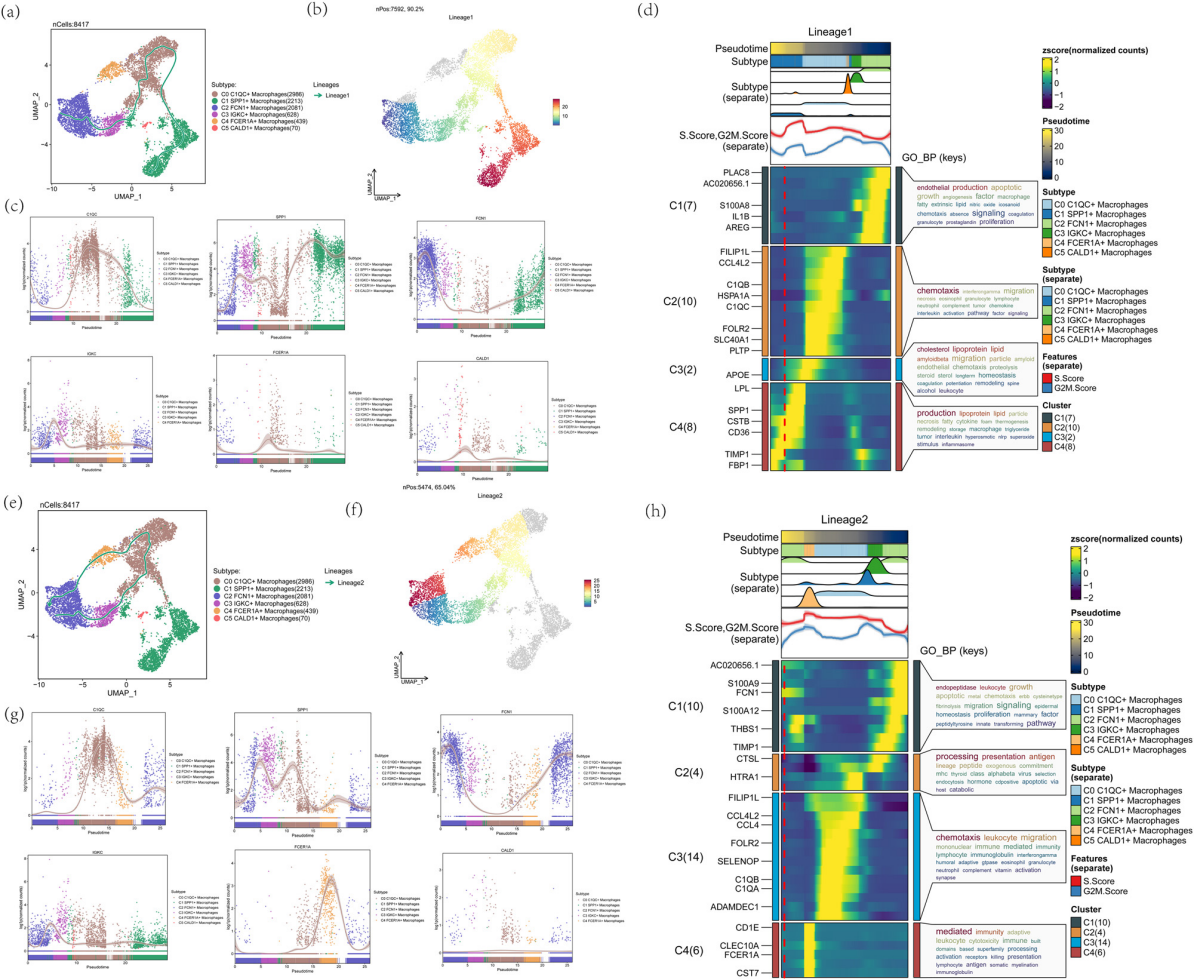
Slingshot analysis of cell trajectories of atherosclerotic macrophages. (a and b) Atherosclerotic macrophages were analyzed using Slingshot to fit and two different differentiation trajectories, Lineage1:C2 FCN1+ macrophages → C3 IGKC+ macrophages → C0 C1QC+ macrophages → C1 SPP1+ macrophages. (c) Proposed time-series scatter plot showing the distribution of maker genes in Lineage1 for each of the six macrophage species. (d) Heatmap showing the analysis results of GOBP enrichment entries based on the proposed temporal trajectory Lineage1. (e and f) Analysis of atherosclerotic macrophages using Slingshot, the proposed and differentiation trajectory, Lineage2:C2 FCN1+ macrophages → C3 IGKC+ macrophages → C0 C1QC+ macrophages → C4 FCER1A+ macrophages → C2 FCN1+ macrophages. (g) Proposed time-series scatter plot showing the distribution of maker genes in Lineage2 for each of the six macrophage species. (h) Heatmap showing the results of the analysis of GOBP-enriched entries based on the fitted temporal trajectory Lineage2.

To further investigate the heterogeneity of atherosclerotic macrophage subpopulations and to infer the cellular genealogical trajectories and pseudotemporal order of atherosclerotic macrophages, slingshot was used to analyze macrophage differentiation trajectories. We identified two cell lineage trajectories of macrophage subclusters, Lineage1:C2 FCN1+ macrophages → C3 IGKC+ macrophages → C0 C1QC+ macrophages → C1 SPP1+ macrophages (Figure 6a and b) and Lineage2:C2 FCN1+ macrophages → C3 IGKC+ macrophages → C0 C1QC+ macrophages → C4 FCER1A+ macrophages → C2 FCN1+ macrophages (Figure 6e and f). Next, the expression changes and distribution of macrophage maker genes within the fitted two pseudo-temporal trajectories, color-coded by cell type, were demonstrated by a pseudo-scatter plot, respectively (Figure 6c and g). To investigate the GOBP enrichment analysis of macrophages with the fitted temporal trajectories, the two trajectories were analyzed separately using tradeSeq. Lineage1 enrichment analysis showed that C2 FCN1+ macrophages were enriched in endothelial production, apoptotic growth and angiogenesis factor macrophage; C0 C1QC+ macrophages were enriched in ChemotaxiS interferon gamma migration, necrosis eosinophil granulocyte lymphocyte, neutrophil complement tumor chemokine, and neutrophil complement tumor chemokine, interleukin activation pathway factor signaling and cholesterol lipoprotein lipid, amyloidbeta migration particle amyloid, endothelial chemotaxis proteolysis, steroid sterol long-term homeostasis; and C1 SPP1+ macrophages enriched in production lipoprotein lipid particle, necrosis fatty cytokine foam thermogenesis, remodeling storage macrophage triglyceride, tumor interleukin hyperosmotic nlrp superoxide, and stimulus inflammasome (Figure 6d). Lineage2 enrichment results showed that C2 FCN1+ macrophages were enriched in endopeptidase leukocyte growth, migration signaling, epidermal homeostasis proliferation mammary factor, and peptidyl tyrosine innate transforming pathway; C1 SPP1+ macrophages and C3 IGKC+ macrophages were enriched in processing presentation antigen and lineage peptide exogenous; C0 C1QC+ macrophages were enriched in chemotaxis leukocyte migration, mononuclear immune mediated immunity, humoral adaptive gtpase eosinophil granulocyte, neutrophil complement; C4 FCER1A+ macrophages were enriched in mediated immunity adaptive, leukocyte cytotoxicity immune built, immunoglobulin (Figure 6h). In conclusion, consistent with the Monocle2 results, C2 FCN1+ macrophages were predominantly located in the initial and terminal stages of the two cell differentiation trajectories simulated by Slingshot. This further underscores the significant role of C2 FCN1+ macrophages in the progression of atherosclerotic disease. Consequently, we will now focus on investigating the signaling pathways associated with C2 FCN1+ macrophages.

### 3.8 Cellular communication patterns in atherosclerotic macrophages

**Figure 7:**
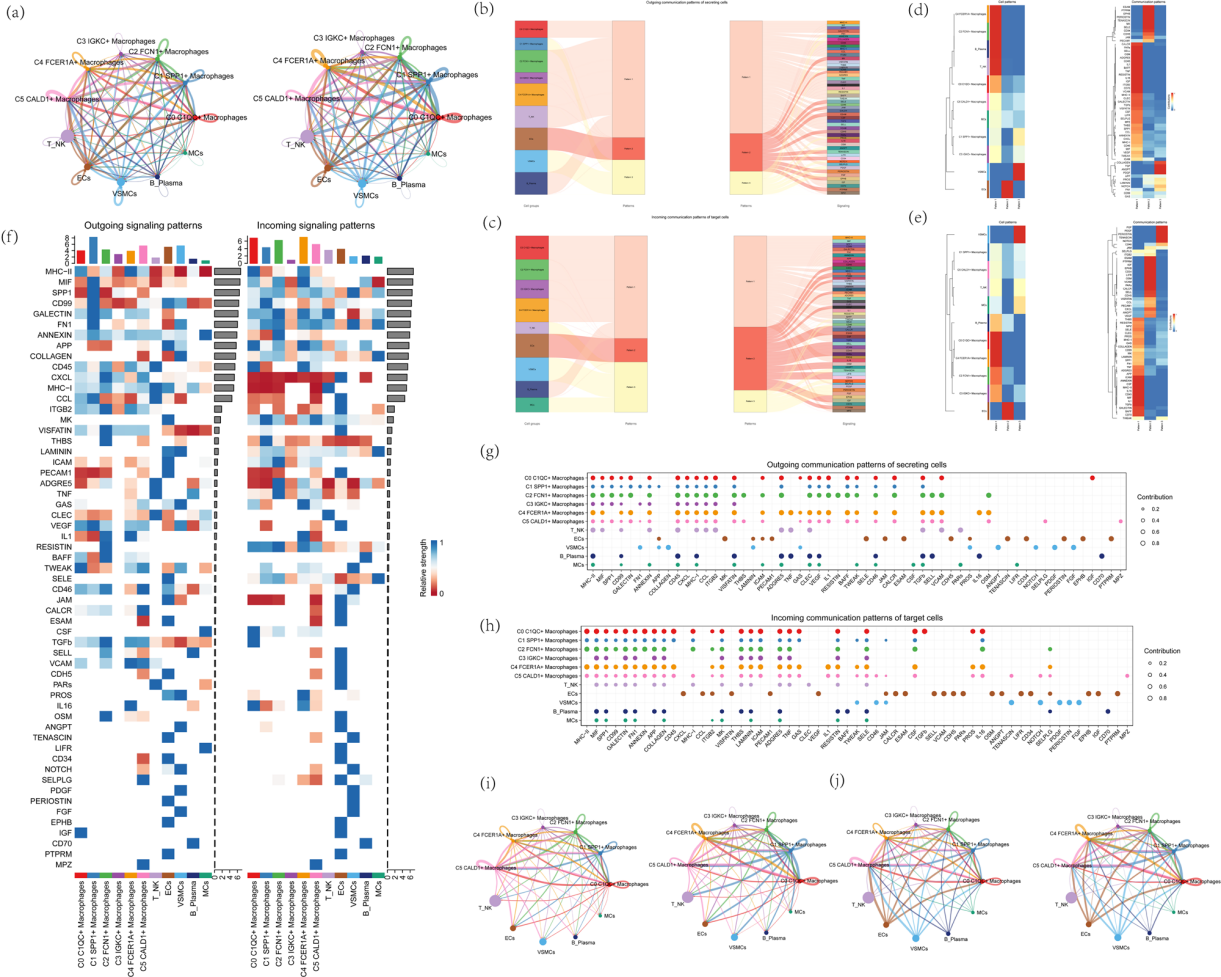
Communication network analysis between atherosclerotic cells. (a) Circle plot demonstrating the number (left) and strength (right) of receptor–ligand interactions in all atherosclerotic cells. (b and d) Afferent signaling patterns of secretory cells (top) and afferent signaling patterns of target cells (bottom) visualized by Sankey diagrams showing the correspondence between inferred potential patterns and cell populations as well as signaling pathways. The thickness of the flux indicates the contribution of the cell population or signaling pathway to each potential pattern. The height of each pattern was proportional to the number of its associated cell population or signaling pathway. Efferent patterns reveal how sending cells coordinate with each other and how they coordinate with specific signaling pathways to drive communication. Afferent patterns reveal how target cells coordinate with each other and how they coordinate. (c and e) Heatmap visualizing pattern recognition of all cellular interactions in atherosclerosis, showing three cases of pattern recognition for afferent signaling and three cases of pattern recognition for efferent signaling. (f) Heatmap showing afferent and efferent signal strengths for all cellular interactions in atherosclerosis. (g and h) To illustrate the afferent communication pattern of target cells and the efferent signaling communication pattern of secretory cells, bubble diagrams were used to show the strength of receptor–ligand interactions. Different colors were used to distinguish different cell types, while the size of the dots was adjusted to indicate the number of cells. (i) Screening for macrophages as a source of cellular interactions, utilizing a circle graph to show the number and intensity of macrophage interactions with other cells. (j) Screening macrophages as targets of cellular interactions, utilizing circle diagrams to demonstrate the number and intensity of macrophage interactions with other cells.

To infer and analyze the communication network between atherosclerotic macrophages, network analysis and pattern recognition methods were used through CellChat software to predict the major signaling inputs and outputs of the cells and how these cells and signals coordinate their functions. Circle plots demonstrate the strength and number of receptor–ligand interactions between atherosclerotic macrophages and all other cells, with relatively strong interactions between macrophages and endothelial cells (Figure 7a). To study the coordinated function of multiple cell populations and signaling pathways, CellChat was used to identify global communication patterns, as well as key signals in different cell groups, outputting a set of communication patterns that supposedly connect cell populations to signaling pathways in the context of efferent signaling (i.e., cells were considered as senders) or afferent signaling (i.e., cells were considered as receivers). The afferent and efferent signaling patterns of the secreted cells were visualized by heatmap and shockmap alluvial plot (Figure 7b, c and d, e), revealing three patterns for efferent signaling (Figure 7b and d) and three for afferent signaling patterns (Figure 7c and e). For example, this output shows that all efferent macrophage signaling was characterized by pattern #1, which represents multiple pathways including, but not limited to, MHC-II, MIF, SPP1, CCL, and CXCL. All efferent endothelial cell signaling was characterized by pattern #2, which represents pathways such as MK, PECAM1, and ESAM. On the other hand, the communication patterns of the target cells indicated that all afferent macrophage signaling was dominated by three modes #1, which included signaling pathways such as CD99, SPP1, and MIF. All afferent endothelial cell signaling was characterized by mode #2, driven by pathways such as CXCL, CCL, and ITGB2. These results suggest that (1) different cell types in the same tissue can rely on signaling networks that overlap to a large extent and (2) certain cell types, such as macrophages, activate multiple signaling modes and pathways at the same time, whereas others, such as endothelial cells, rely on fewer and more homogeneous communication patterns. Next, to illustrate the afferent communication patterns of target cells and the efferent signaling communication patterns of secretory cells, bubble plots and heatmaps were used to demonstrate the strength of receptor–ligand interactions (Figure 7f–h). Finally, to further investigate the interaction between macrophages and other cells, macrophages were screened as the source of cellular interactions, and circle diagrams were used to demonstrate the number and intensity of macrophage–other cell interactions (Figure 7i); macrophages were screened as the target of cellular interactions, and circle diagrams were used to demonstrate the number and intensity of macrophage–other cell interactions (Figure 7j).

### 3.9 Signaling patterns of CCL and amyloid precursor protein (APP) signaling pathways

**Figure 8:**
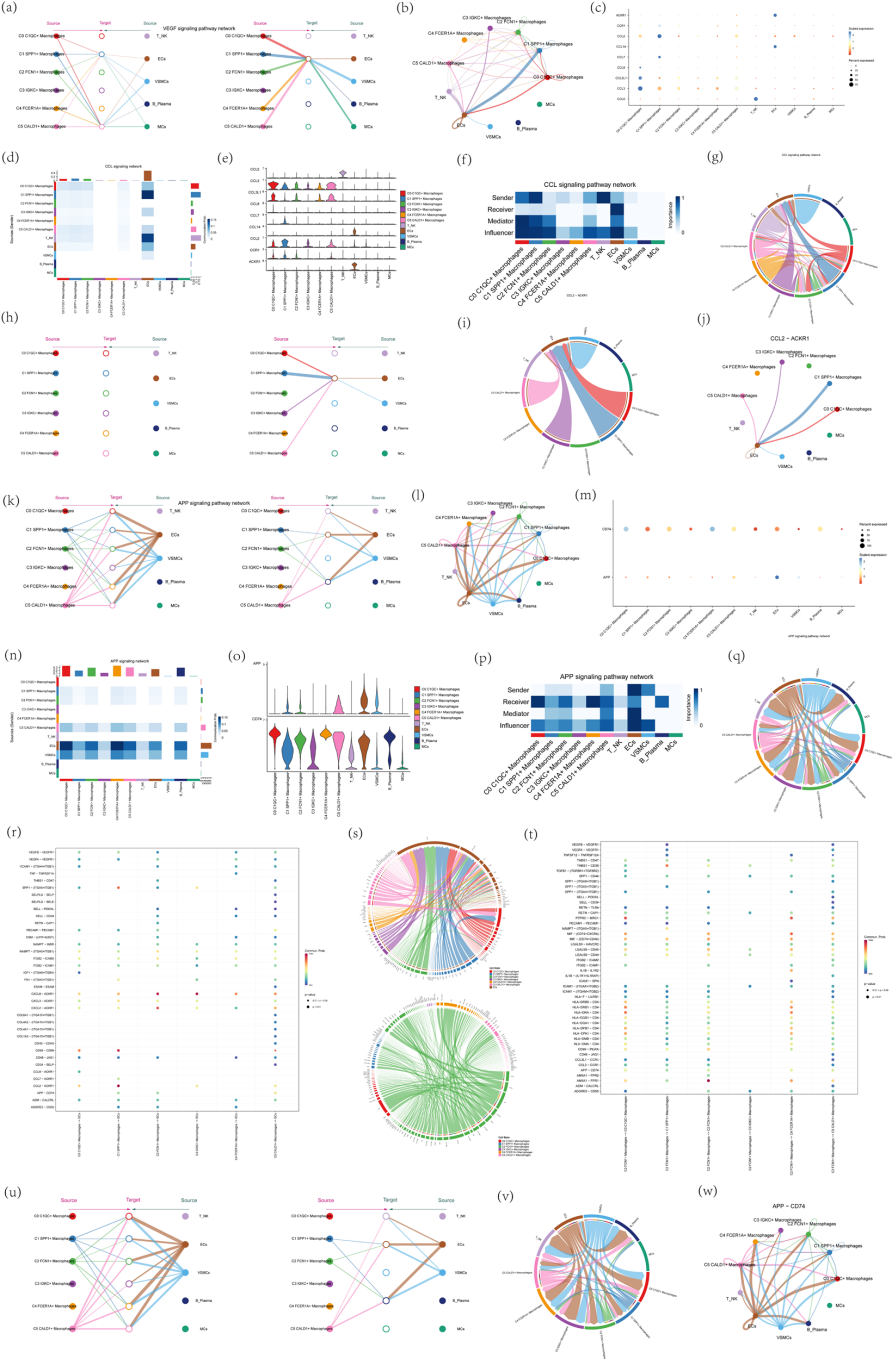
Screening signaling pathways for strong interactions between macrophages and endothelial cells. (a–c) Screening all macrophages as the SOURCE, endothelial cells as the TARGET, and the signaling pathway with the strongest interaction between cells as the CCL signaling pathway. Use the layered diagram, circle diagram, and bubble diagram to demonstrate the intercellular communication network conducted by the CCL signaling pathway. The layered diagram consists of two parts: the left part and the right part highlight autocrine and paracrine signaling in the macrophage state and other cell states, respectively. (d, e, and g) Heatmaps, violin diagrams, and chord diagrams of cellular interactions of the CCL signaling pathway. (f) Heatmap based on four network centrality measures of the CCL signaling network showing the relative importance of each cell group. (h–j) Hierarchical, chordal, and circle diagrams demonstrating the intercellular communication network with CCL2–ACKR1 as the receptor–ligand pair interaction. (k–m) Screening of all endothelial cells as the SOURCE, all macrophages as the TARGET, and the signaling pathway with the strongest intercellular interactions as the APP signaling pathway. Use hierarchical diagrams, circle diagrams, and bubble diagrams to demonstrate the intercellular communication network for APP signaling pathway transduction. (n, o, and q) Heatmaps, violin plots, and chord plots of APP signaling pathway cell interactions. (p) Heatmap based on four network centrality measures of the APP signaling network showing the relative importance of each cell group. (r) Screening endothelial cells as the SOURCE and C2 FCN1+ macrophage subpopulation as the TARGET, the signaling pathway with the strongest cellular interactions is also the APP signaling pathway. Intercellular receptor–ligand interaction dot plots when screening endothelial cells as source. (s) Chordal diagram of receptor–ligand interactions when screening endothelial cells as source (top) and C2 FCN1+ macrophage subpopulation as target (bottom). (t) Dot plot of inter-cellular receptor–ligand interactions when screening a subpopulation of C2 FCN1+ macrophages as target. (u–w) Hierarchical, chordal, and circle diagrams demonstrating the intercellular communication network with APP-CD74 as the receptor–ligand pair interaction.

To further investigate the interactions between macrophages and endothelial cells, important cellular interaction pathways were identified. We screened the pathways of macrophage–endothelial cell interactions for study and screened all macrophages as the SOURCE and endothelial cells as the TARGET, the signaling pathway with the strongest cellular interactions was the CCL signaling pathway, and the inter-cellular communication network transmitted by the CCL signaling pathway was shown in Figure 8a–g. Notably, the CCL ligand–receptor pair, i.e., CCL2–ACKR1, was the major contributor to this communication network, and the specifics of CCL2–ACKR1 receptor–ligand interactions were shown in Figure 8h–j. Next, screening all endothelial cells as the SOURCE and all macrophages as the TARGET, the signaling pathway with the strongest intercellular interactions was the APP signaling pathway, and the intercellular communication network conducted by the APP signaling pathway was shown in Figure 8k–q. The results of monocle2 and Slingshot proposed timing analysis found that C2 FCN1+ macrophages were at the end of the differentiation trajectory and C2 FCN1+ macrophages were still at the initial stage of the trajectory in Slingshot’s Lineage2, which suggests that there was a very important role with the progression of atherosclerotic disease. Therefore, we screened ECs as source and C2 FCN1+ macrophages as target, and the signaling pathway with the strongest cell–cell interaction was also the APP signaling pathway. The APP ligand–receptor pair, i.e., APP-CD74, was the main contributor to this communication network, and the specifics of APP-CD74 receptor–ligand interactions were shown in Figure 8u–w. Intercellular receptor–ligand interactions when endothelial cells were screened as source and C2 FCN1+ macrophage subpopulation as target were shown in Figure 8r–t.

### 3.10 Visualization of CXCL signaling pathway

**Figure 9:**
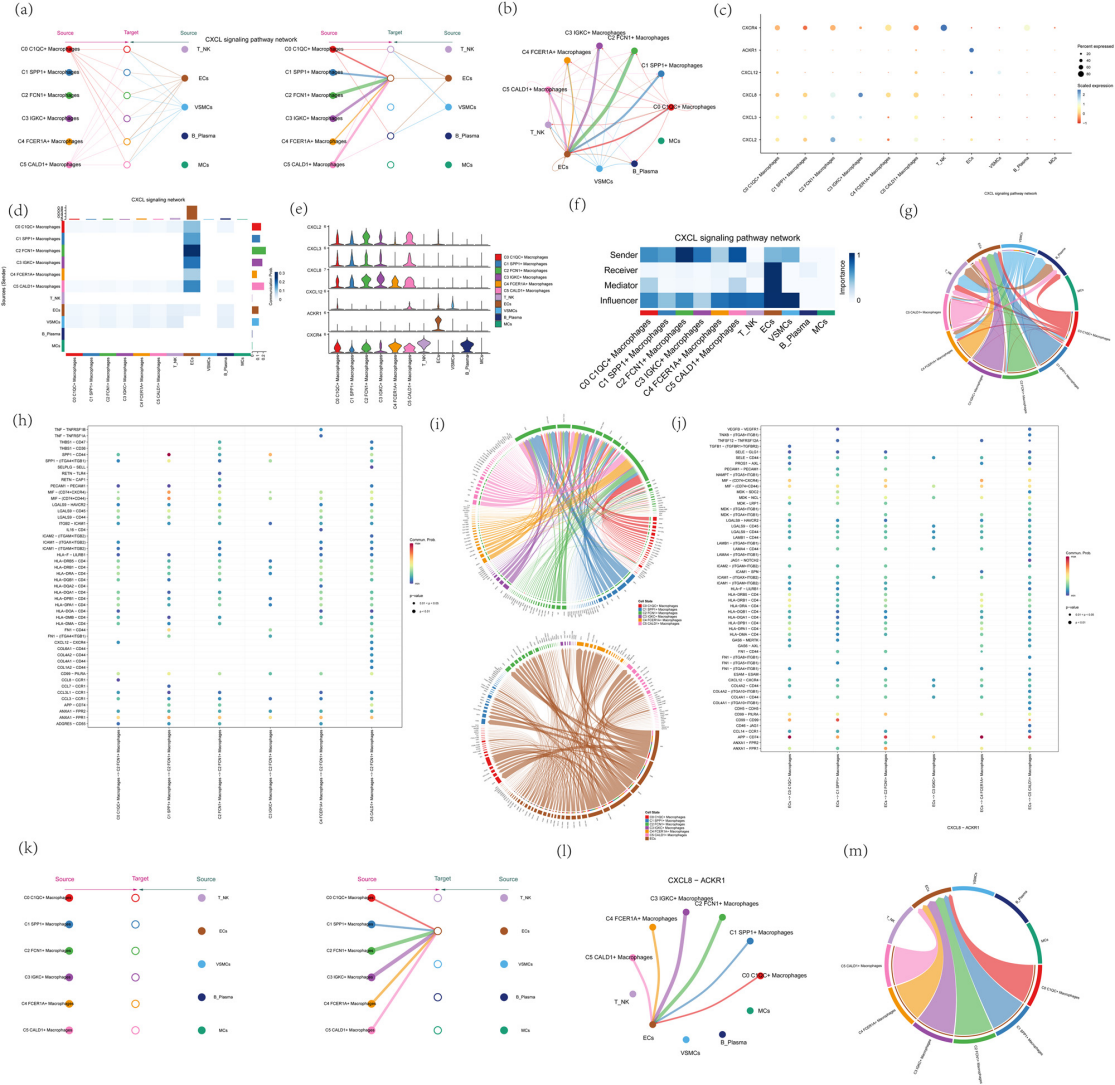
Visual analysis of CXCL signaling pathway. (a–c) Screening C2 FCN1+ macrophage subpopulation as source and endothelial cells as target, the signaling pathway with the strongest cell-to-cell interaction was the CXCL signaling pathway. Use hierarchical diagrams, circle diagrams, and bubble diagrams to demonstrate the intercellular communication network conducted by the CXCL signaling pathway. (d, e, g) Heatmap, violin diagram, and chord diagram of CXCL signaling pathway cell interactions. (f) Heatmap based on four network centrality measures of the CXCL signaling network showing the relative importance of each cell group. (h) Screening of C2 FCN1+ macrophage subpopulation as SOURCE, for endothelial cell TARGET, the signaling pathway with the strongest cellular interactions is also the APP signaling pathway. Dot plot of intercellular receptor–ligand interactions when screening C2 FCN1+ macrophage subpopulation for source. (i) Chordal diagram of receptor–ligand interactions when screening C2 FCN1+ macrophage subpopulation as source (top) and endothelial cells as target (bottom). (j) Dot plot of intercellular receptor–ligand interactions when screening endothelial cells as target. (k–m) Hierarchical, chordal, and circle diagrams showing the intercellular communication network with CXCL8–ACKR1 as the receptor–ligand pair interaction.

Screening the C2 FCN1+ macrophage subpopulation as the source and endothelial cells as the target, the signaling pathway with the strongest intercellular interactions was the CXCL signaling pathway, and the intercellular communication network transmitted by the CCL signaling pathway was shown in Figure 9a–g. Screening all C2 FCN1+ macrophages as the SOURCE and all endothelial cells as the TARGET, the signaling pathway with the strongest cell-to-cell interaction was the CXCL signaling pathway. Intercellular receptor–ligand interactions when screening a subpopulation of C2 FCN1+ macrophages as source and a subpopulation of endothelial cells as target were shown in Figure 9h–j. Notably, the CXCL ligand–receptor pair, i.e., CXCL8–ACKR1, was the major contributor to this communication network, and the specifics of CXCL8–ACKR1 receptor–ligand interactions were shown in Figure 9k–m. CXCL8–ACKR1 receptor–ligand interactions were shown in Figure 9k–m.

## 4 Discussion

Atherosclerotic CVD is a lipid-driven chronic inflammatory disease caused by maladaptive immune responses resulting from imbalances in lipid metabolism and accumulation of cholesterol-rich macrophages in the arterial wall [61], where macrophages are responsible for the uptake of these lipids and drive disease progression. Normal arterial walls contain endothelial cells, smooth muscle cells (SMCs), epithelial fibroblasts, vascular macrophages [17], T cells, and B cells [25,63]. The atherosclerotic cell microenvironment is altered, which activates endothelial cells, and changes the SMC phenotype [64,65] and local proliferation of vascular macrophages [66,67]. Stem cells were observed in the outer membrane to potentially give rise to SMCs and macrophages [66,68]. The occurrence and development of atherosclerosis depend on local inflammation and lipid accumulation in the vessel wall. In each vascular bed, macrophages contribute to the maintenance of the local inflammatory response, propagate plaque development, and promote thrombosis [69]. In atherosclerotic lesions, macrophages are fundamental contributors, and macrophages receive multiple microenvironmental signals, such as oxidized lipids and cytokines, which affect macrophage phenotypic polarization and activation, resulting in dynamic plasticity [70]. In atherosclerosis, macrophages play a central role in the formation, growth, and eventual rupture of atheromatous plaques, and metabolic alterations are a key feature in determining macrophage function and subsequent disease progression [71]. Lesion macrophages can perform a number of different functions to control the development of atherosclerotic lesions, and it has long been recognized that plaques contain several phenotypically heterogeneous macrophage subpopulations [71]. However, the methodology used to define the identity of these macrophages appears to be inherently biased because *in vitro* macrophage stimulation from a single source using a limited range of specific stimuli does not reflect the identity of the macrophage [72,73]. Pathogenic macrophages exhibit different phenotypes, depending on their environment and activated intracellular signaling pathways. These different phenotypes enable macrophages to phagocyte lipids, dead cells, and other substances that are considered danger signals.

The occurrence and development of atherosclerosis depend on local inflammation and lipid accumulation in the vessel wall. Macrophages are the most fundamental contributors involved in the occurrence and development of atherosclerosis. Macrophages are affected by a variety of microenvironmental signals such as oxidized lipids and cytokines, which affect the phenotype polarization and activation of macrophages in the process of atherosclerotic lesions [74]. Macrophages have heterogeneity and dynamic plasticity. M1 macrophages primarily drive inflammatory responses and disease progression in atherosclerosis through the secretion of pro-inflammatory cytokines (such as IL-1β, IL-6, and TNF-α) and the generation of reactive oxygen species (ROS) [70]. In contrast, M2 macrophages contribute to atherosclerosis by secreting anti-inflammatory factors (such as IL-10 and TGF-β), promoting fibrosis and plaque stabilization, thereby reducing inflammation and facilitating tissue repair [75]. In addition, the study of macrophage heterogeneity may guide new therapeutic targets against cardiovascular diseases such as atherosclerosis [70,76]. Through the utilization of single-cell analysis, we can delve into comprehending the functionality and interactions of distinct cell types during the developmental processes of atherosclerosis. The disparities and interplay among these cells have the potential to unveil novel therapeutic targets, thereby establishing a foundation for the development of more precise and personalized treatment strategies. By identifying and targeting disease-relevant signaling pathways specific to particular cellular subpopulations, we can intervene in the progression of the disease with greater specificity, thereby augmenting treatment efficacy.

The advent of single-cell RNA sequencing presents an opportunity to profile and identify cellular identities in an unbiased manner, without relying on predefined labeling strategies. This technique enables the exploration of previously unknown cell populations or functional states associated with diseases, along with the discovery of their markers and potential molecular regulators. Single-cell technologies, as indicated by studies [77,78], have the potential to enhance our comprehension of the progression of atherosclerotic disease and provide valuable insights for developing novel diagnostic and therapeutic approaches aimed at mitigating the risk of cardiovascular disease. Given their significant involvement in the advancement of atherosclerosis, macrophages represent a crucial focus for diagnostic imaging and the development of innovative therapies to address the pathological processes associated with macrophages in atherosclerosis.

Furthermore, single-cell analysis also contributes to our understanding of treatment efficacy and prognostic predictions. By monitoring the variations in cell types and transcriptomes following therapeutic interventions, we can evaluate treatment responses and make individualized treatment adjustments based on this information. This precise therapeutic approach holds the promise of improving patient prognoses and reducing unnecessary treatments. The translation of these findings into clinical applications necessitates further research and collaboration. Through close cooperation with clinicians and researchers, we can integrate the results of single-cell analysis with clinical characteristics, leading to the development of more accurate diagnostic tools and treatment strategies. Additionally, the establishment of large-scale single-cell databases is imperative to validate and corroborate the novel discoveries with clinical data.

Through a series of single-cell analyses, the selected atherosclerotic macrophages were classified into six distinct subpopulations. Based on the marker genes of these various cells, these refined macrophage clusters were categorized into six types: C0 C1QC+ macrophages, C1 SPP1+ macrophages, C2 FCN1+ macrophages, C3 IGKC+ macrophages, C4 FCER1A+ macrophages, and C5 CALD1+ macrophages. SPP1 (osteopontin) is a crucial extracellular matrix protein involved in cell adhesion, migration, and inflammation regulation. SPP1+ macrophages are commonly associated with plaque instability and lesion progression in atherosclerotic plaques [79]. FCN1 (mannose-binding lectin 1) plays a critical role in innate immunity by binding to pathogens and facilitating their phagocytosis. FCN1+ macrophages play a pivotal role in anti-inflammatory responses and tissue repair [80]. The results of Monocle2 and Slingshot cell trajectory inference are consistent: C1 SPP1+ macrophages mostly in state1, C0 C1QC+ macrophages mostly in state5, C2 FCN1+ macrophages, C3 IGKC+ macrophages mostly in state3 and state4, C4 FCER1A+ macrophages, and C5 CALD1+ macrophages mostly in state2 and state3; it can be roughly inferred that atherosclerotic macrophages undergo an evolution from C1 SPP1+ macrophages to C4 FCER1A+ macrophages, C5 CALD1+ macrophages, then to C2 FCN1+ macrophages, C3 IGKC+ macrophages, and finally, to C0 C1QC+ macrophages. Macrophage differentiation trajectories were analyzed using slingshot. We found two cell lineage trajectories of macrophage subclusters, Lineage1:C2 FCN1+ macrophages → C3 IGKC+ macrophages → C0 C1QC+ macrophages → C1 SPP1+ macrophages and Lineage2:C2 FCN1+ macrophages → C3 IGKC+ macrophages → C0 C1QC+ macrophages → C4 FCER1A+ macrophages → C2 FCN1+ macrophages. The above results indicate that the C2 FCN1+ macrophages subpopulation plays an important role in the progression of atherosclerosis and may provide a new clinical diagnosis, atherosclerosis progression, providing new clinical targets. In previous studies, the activation trajectory of the macrophage subtypes was divided into three states (Prebranch, Cell Fate 1, and Cell Fate 2), and Cell Fate 2 domined in SPP1+/VCAN+ macrophages, IL1B+ macrophages, and FLT3LG+ macrophages, with distinct interactions with other cell components and relating to a poorer prognosis of ischemic events [81]. According to pseudotime analysis, M2/M1 macrophages and M2 macrophages can be transformed into M1 macrophages [82]. Utilizing analytical tools such as Monocle2 and Slingshot, the heterogeneity and phenotypic transformation of macrophages in atherosclerosis have been explored in depth. These analyses elucidate how macrophage transition from inflammatory states to anti-inflammatory or fibrotic states during disease progression, offering insights into the potential impacts of these transformations on disease development. Such studies contribute to a deeper understanding of macrophage roles in atherosclerosis and provide a foundation for developing targeted therapeutic strategies.

To understand the enrichment of genes in the gene set of atherosclerosis macrophages subpopulation in terms of BP, MF, and cell composition, we analyzed the GOBP enrichment analysis results of six macrophage differential genes. In the enrichment analysis, the most significant findings include that C1 SPP1+ macrophages are notably enriched in the following processes: purine ribonucleoside triphosphate metabolic process, purine nucleoside triphosphate metabolic process, ribonucleoside triphosphate metabolic process, oxidative phosphorylation, and nucleoside triphosphate metabolic process. Subsequent *in vitro* experiments revealed that Psap inhibition suppressed both glycolysis and oxidative phosphorylation [83]. Ox-LDL could translocate Gsα from macrophage lipid rafts in the short term and promote Gnas transcription through ERK1/2 activation and C/EBPβ phosphorylation via oxidative stress in the long term [84]. C2 FCN1+ macrophages are enriched in cytoplasmic translation, phagocytosis, positive regulation of cytokine production, leukocyte proliferation, and positive regulation of response to external stimulus. Phagocytosis of polymeric nanoparticles aided activation of macrophages to increase atherosclerotic plaques in ApoE−/− mice [85]. Clearance of apoptotic cells (ACs) by phagocytes (efferocytosis) prevents post-apoptotic necrosis and dampens inflammation. Defective efferocytosis drives important diseases, including atherosclerosis. For efficient efferocytosis, phagocytes must be able to internalize multiple ACs [86].

Atherosclerosis is a complex chronic inflammatory process involving multiple interactions between endothelial cells and macrophages. The initial stage of atherosclerosis typically begins with endothelial cell damage or dysfunction [87]. Damaged endothelial cells release various inflammatory factors that recruit and activate immune cells, such as macrophages, from the bloodstream to the site of injury [88]. Endothelial dysfunction leads to LDL cholesterol buildup and its oxidation. Both endothelial cells and macrophages take up oxidized LDL, speeding up atherosclerosis [89]. Macrophages also release inflammatory substances, worsening inflammation and plaque growth. Overall, endothelial cells and macrophages are key in atherosclerosis development, and targeting their functions could help prevent or treat it [90]. The endothelium serves as the origin of mesenchymal cells associated with plaque formation. Endothelial cells have the ability to undergo endothelial-mesenchymal transition (EndMT), wherein they lose their characteristic endothelial cell markers and functions while gaining the expression of mesenchymal cell markers and functions [91]. Furthermore, EndMT triggers the stratification and migration of mesenchymal cells derived from the endothelium into the underlying tissue [91]. Studying the interactions between macrophages and endothelial cells identifies important cellular interaction pathways. We screened the pathways of macrophage-endothelial cell interactions for study and screened all macrophages as the SOURCE and endothelial cells as the TARGET, and the signaling pathway with the strongest cellular interactions was the CCL signaling pathway, and the CCL ligand–receptor pairs, i.e., CCL2–ACKR1, were the major contributors to this communication network. Research on atherosclerotic disease has demonstrated the significant role of the CCL signaling pathway. The CCL signaling pathway, particularly involving the CCL5–CCR1 and CCL5–CCR5 pairs, is crucial in T cells [92]. In atherosclerotic plaques from P2X4-deficient mice, we observed reduced RNA expression of several inflammatory markers, including CCL-2, CXCL-1, CXCL-2, IL-6, and TNFα, as well as decreased NLRP3-inflammasome priming [93]. Additionally, bone marrow-derived macrophages from these mice showed lower ATP-induced release of CCL-2, CCL-5, IL-1β, and IL-6. Although ELR(+)-CXCL chemokines are known for their role in acute inflammation by attracting neutrophils, their involvement in chronic atherosclerosis is less understood. In our mouse model of atherosclerosis, CXCL5 expression increased during disease progression, but this was not linked to neutrophil infiltration. Next, all endothelial cells were screened as the SOURCE, all macrophages as the TARGET, and the signaling pathway with the strongest cell-to-cell interactions was the APP signaling pathway. The APP ligand–receptor pair, i.e., APP-CD74, was the major contributor to this communication network. The results of monocle2 and Slingshot proposed time-series analysis found that C2 FCN1+ macrophages were at the end of the differentiation trajectory, and C2 FCN1+ macrophages were still at the initial stage of the trajectory in Slingshot’s Lineage2, suggesting that there is a very important role with the progression of atherosclerotic disease. Therefore, we screened ECs as the source, C2 FCN1+ macrophages as the target, and the signaling pathway with the strongest cell-to-cell interaction was also the APP signaling pathway. APP is a ubiquitously expressed type 1 integral membrane protein. In the cerebrovascular systems of tissues affected by atherosclerosis and Alzheimer’s disease (AD), the protein levels of APP, phosphorylated APP (Tyr682), and β-amyloid (Aβ) are elevated [94].

Future research should integrate single-cell RNA sequencing with proteomics data to comprehensively understand the transcriptomic and proteomic profiles of macrophage subpopulations [94]. This integration will help elucidate the unique functional states of these subpopulations and their contributions to atherosclerosis. Metabolomic analyses of macrophage subpopulations can further clarify how these cells participate in atherosclerosis through metabolic pathways [95]. For instance, studying the roles of macrophage subpopulations in lipid metabolism and inflammatory responses may identify novel metabolic biomarkers and intervention targets. Employing systems biology approaches to integrate genomics, transcriptomics, proteomics, and metabolomics data will enable the construction of functional network models of macrophage subpopulations in atherosclerosis [96]. This will facilitate a systematic understanding of interactions between different subpopulations and their collective impact on disease progression. Based on macrophage subpopulation characteristics, new biomarkers can be developed for early diagnosis and disease staging of atherosclerosis, potentially enhancing diagnostic accuracy and enabling personalized medicine. Evaluating disease progression and risk using macrophage subpopulation characteristics can inform individualized treatment plans and prognostic predictions. New drugs or immunotherapy strategies targeting specific macrophage subpopulations could be developed to slow or reverse the progression of atherosclerosis, focusing on molecules or signaling pathways unique to these subpopulations [97]. By analyzing macrophage subpopulation characteristics, personalized treatment plans tailored to individual patients’ specific subpopulation features can be devised. In-depth studies of macrophage subpopulations in atherosclerosis, especially through the integration of multi-omics data and the development of novel experimental models, will offer new insights into their mechanisms. These studies will have significant clinical implications, including improved diagnostic methods, prognostic assessments, and therapeutic strategies, ultimately enhancing the management of atherosclerosis and patient quality of life.

In conclusion, we identified the key macrophage subpopulation C2 FCN1+ macrophages associated with atherosclerosis by using a series of methods of single-cell analysis and interacted with endothelial cells through CCL, CXCL, APP, and other pathways to regulate the progression of atherosclerosis. Research on atherosclerotic disease has demonstrated the significant role of the CCL signaling pathway.

## 5 Conclusion

The differentiation process of macrophages in atherosclerosis was studied by characterizing the single-cell landscape of atherosclerosis. A series of single-cell sequencing analysis methods were used to infer the cell trajectory and identify the key macrophage subsets C2 FCN1+ macrophages associated with atherosclerosis, and interact with endothelial cells through CCL, CXCL, APP, and other pathways. The key macrophage subset could regulate the progression of atherosclerosis. By studying the cellular function of key macrophage subsets and revealing key pathways in the progression of atherosclerosis, we hope to provide new therapeutic targets for better clinical guidance, while also playing an important role in single-cell technology research in the field of cardiovascular disease.

## Supplementary Material

Supplementary Figure
