# Supplementary material for: Single-cell analysis identified key macrophage subpopulations associated with atherosclerosis
Source: Open Med (Wars). 2024 Dec 20;19(1):20241088. doi: 10.1515/med-2024-1088 (PMC11669903; doi:10.1515/med-2024-1088)
Supplement: Supplementary Figure [file med-2024-1088-sm.pdf]

Supplementary material

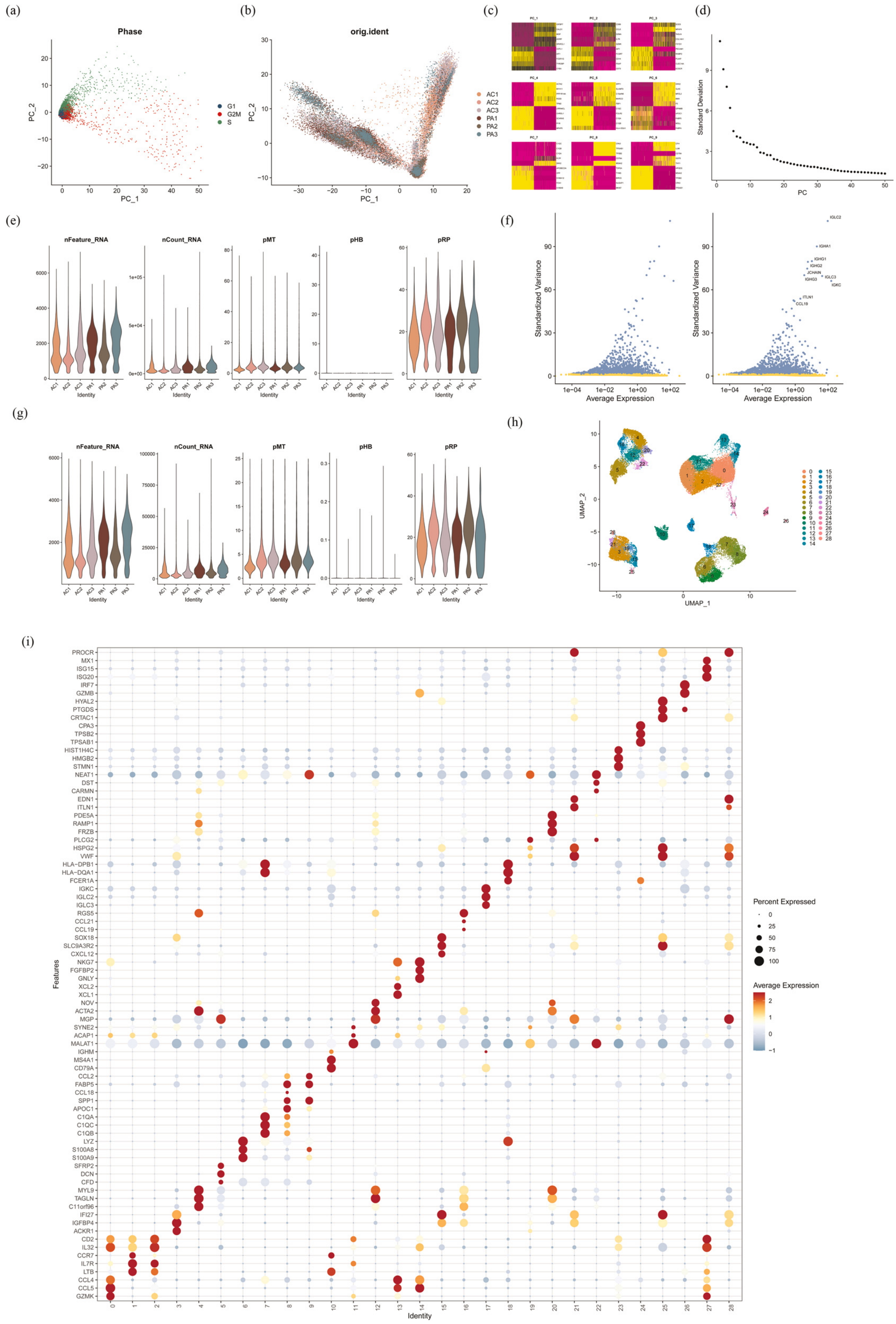

Figure S1: Quality control and screening. (a)–(d) scatter plots demonstrating the results of principal component analysis of atherosclerotic cells. (e) Violin plot demonstrating n Feature_RNA, n Count RNA, pMT, pHB, pRP levels of atherosclerotic cells before quality control. (f) Volcano plot demonstrating differential expression of highly variable genes. (g) Violin plot demonstrating atherosclerotic cell n Feature_RNA, n Count RNA, pMT, pHB, pRP levels after quality control. (h) UMAP plot demonstrating clustering results of cells. (i) Bubble plot demonstrating clustering into 29 cell clusters based on differential gene expression.

(a)

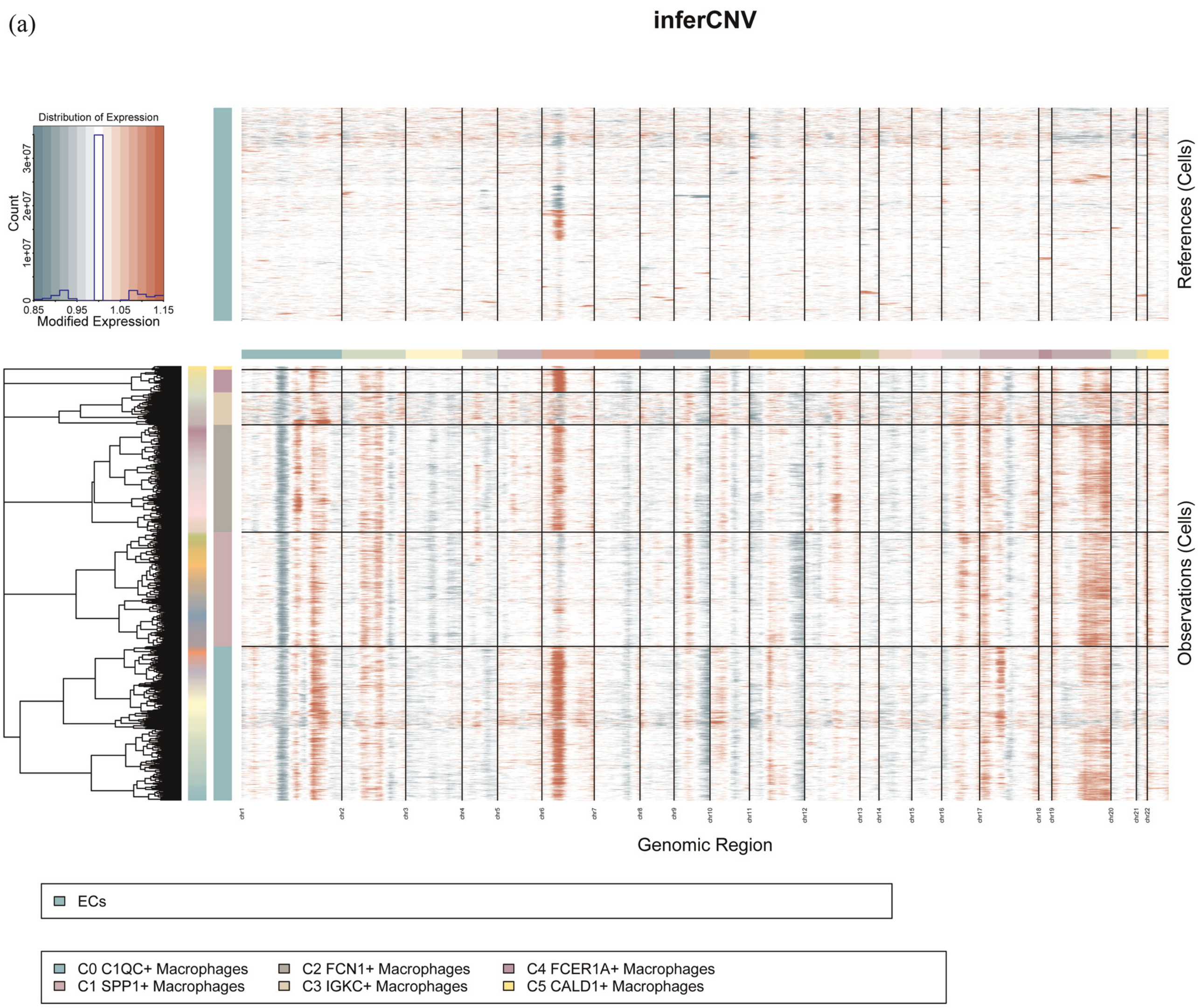

Figure S2: Infer CNV analysis results. (a) Heatmap showing the chromosomal landscape of inferred large-scale copy number variants (inferCNVs). Endothelial cell-based scRNA-seq was used to infer copy number variation in chromosomes of macrophage types in 6. Red color indicates increased copy number variation and green color indicates decreased copy number variation.

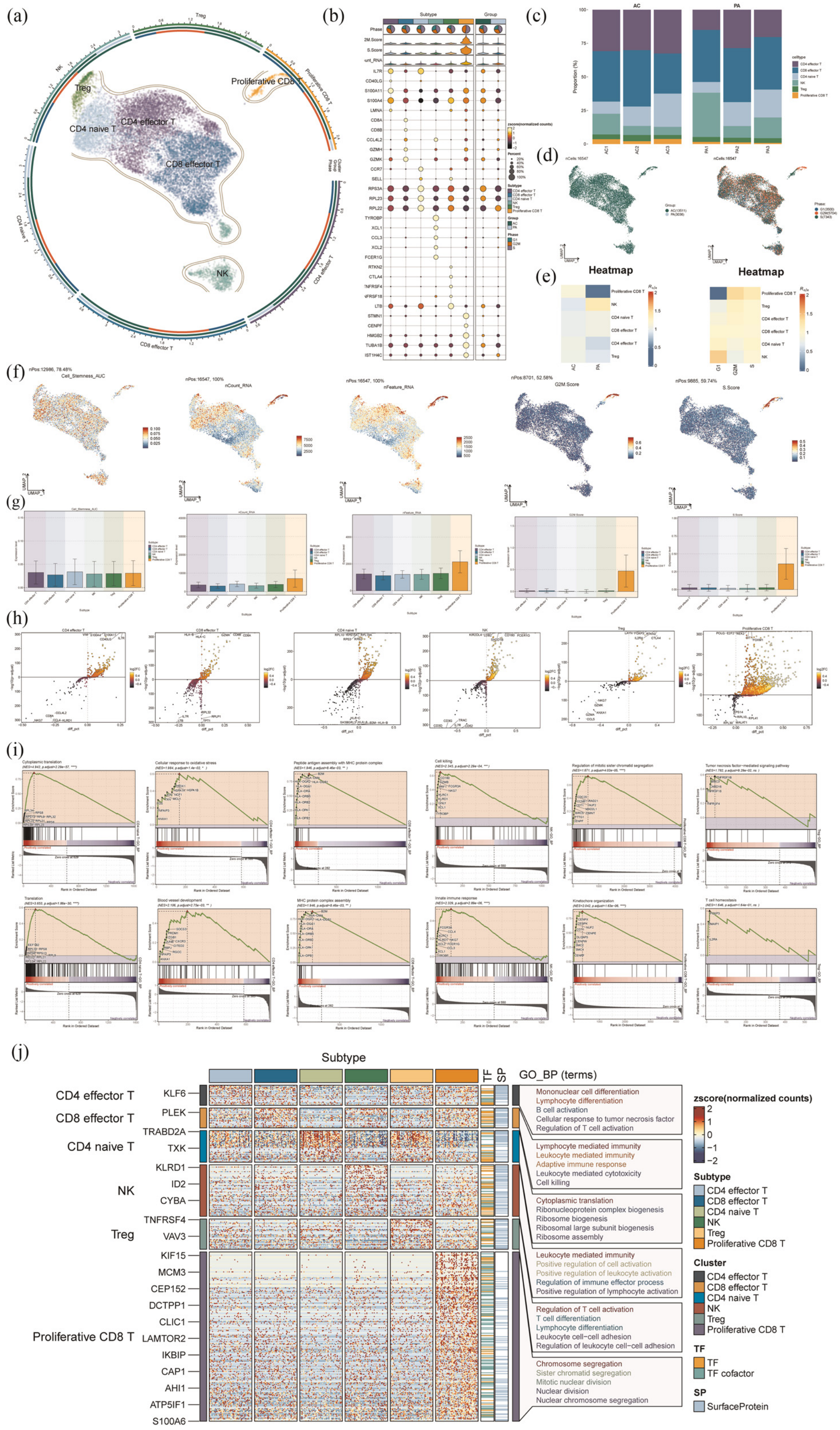

Figure S3: The single-cell landscape of T cell subpopulations. (a) A circular diagram illustrates six T and NK cell subpopulations in atherosclerosis. (b) A bubble plot depicts the expression levels of the top five differentially expressed genes among the six cell subpopulations. (c) A stacked bar chart presents the tissue origin proportions of T cell and NK cell subpopulations. (d) and (e) UMAP plots and heatmaps reveal the tissue origins and cell cycle distributions of T cell and NK cell subpopulations. (f) and (g) UMAP plots and bar graphs display the scores for Cell_Stemness_AUC, nCount_RNA, nFeature_RNA, G2M.Score, and S.Score across the six cell subpopulations. (h) A volcano plot illustrates the upregulated and downregulated genes among the differentially expressed genes of the six cell subpopulations. (i) GSEA results for positive enrichment analysis of T cell and NK cell subpopulations. (j) Enrichment analysis results of differentially expressed genes from the six cell subpopulations within GOBP categories.
